# Supplementary material for: Social corrections act as a double-edged sword by reducing the perceived accuracy of false and real news in the UK, Germany, and Italy
Source: Commun Psychol. 2024 Feb 13;2:10. doi: 10.1038/s44271-024-00057-w (PMC11332053; doi:10.1038/s44271-024-00057-w)
Supplement: Supplementary file 2 — Supplementary Material [file 44271_2024_57_MOESM2_ESM.pdf]

Supplementary Information

Social corrections act as a double edged sword by reducing the perceived accuracy of false and real news in the UK, Germany, and Italy.

Florian Stoeckel[1†*], Sabrina Stöckli[2,3†], Besir Ceka[4],

Chiara Ricchi[1], Ben Lyons[5], and Jason Reifler[1]

[1] Department of Politics, University of Exeter, Exeter, UK

[2] Department of Consumer Behavior, University of Berne, Berne, Switzerland

[3] Department of Business Administration, University of Zurich, Zurich, Switzerland

[4] Department of Political Science, Davidson College, USA

[5] Department of Communication, University of Utah, Salt Lake City, UT, USA

*Corresponding author(s). E-mail(s): F.Stoeckel@exeter.ac.uk

†These authors contributed equally to this work.

This project received funding from the British Academy (BA Award SRG20\200348).

Supplementary Methods

UK Experiment (Experiment 1)

The following table gives an overview of the detailed results of the interaction of social corrections with anti-expert sentiments ($RQ_{Anti\text{-}expert}$), susceptibility to social influence ($RQ_{Social\ influence}$), and cognitive reflection ($RQ_{Cognitive\ reflection}$).

Supplementary Table 1. The moderating role of anti-expert sentiments ($RQ_{Anti\text{-}expert}$) in the case of false news

Accuracy				
Fixed effects	*B*	*t*	*95% CI*	*p*
(Intercept)	1.52	15.75	1.33 – 1.71	<0.001
Anti-expert sentiments	0.31	14.98	0.27 – 0.35	<0.001
Treatment (low amplification)	-0.27	-4.21	-0.39 – -0.14	<0.001
Treatment (high amplification)	-0.26	-4.16	-0.39 – -0.14	<0.001
Treatment (correction with link)	-0.33	-5.25	-0.46 – -0.21	<0.001
Gender (male)	0.03	1.10	-0.02 – 0.09	0.272
Age (25-34)	-0.01	-0.12	-0.11 – 0.10	0.902
Age (35-44)	-0.09	-1.67	-0.19 – 0.02	0.095
Age (45-54)	-0.35	-6.58	-0.45 – -0.24	<0.001
Age (55-64)	-0.50	-9.23	-0.61 – -0.40	<0.001
Age (65+)	-0.55	-10.66	-0.65 – -0.45	<0.001
Education (technical or vocational degree)	-0.01	-0.15	-0.07 – 0.06	0.884
Education (university degree)	0.04	1.22	-0.02 – 0.11	0.222
Treatment (low amplification):Anti-expert sentiments	0.06	2.78	0.02 – 0.10	0.006
Treatment (high amplification):Anti-expert sentiments	0.05	2.25	0.01 – 0.09	0.025
Treatment (correction with link):Anti-expert sentiments	0.07	3.58	0.03 – 0.12	<0.001
Random effects	*Variance*	*SD*	mR^2	0.21
Participants (intercept)	0.31	0.56	cR^2	0.59
False News Posts (intercept)	0.03	0.19		
Residual	0.37	0.61		
Liking				
Fixed effects	*B*	*t*	*95% CI*	*p*
(Intercept)	0.86	8.05	0.65 – 1.07	<0.001
Anti-expert sentiments	-0.18	-2.82	-0.30 – -0.05	0.005
Treatment (low amplification)	-0.06	-0.92	-0.18 – 0.07	0.358
Treatment (high amplification)	-0.09	-1.43	-0.22 – 0.03	0.152
Treatment (correction with link)	0.56	20.78	0.51 – 0.62	<0.001
Gender (male)	0.09	2.09	0.01 – 0.17	0.037
Age (25-34)	-0.01	-0.12	-0.17 – 0.15	0.902
Age (35-44)	-0.24	-3.06	-0.39 – -0.08	0.002
Age (45-54)	-0.67	-8.73	-0.82 – -0.52	<0.001

Age (55-64)	-0.88	-11.05	-1.03 – -0.72	<0.001
Age (65+)	-0.99	-13.17	-1.14 – -0.84	<0.001
Education (technical or vocational degree)	-0.00	-0.01	-0.10 – 0.10	0.991
Education (university degree)	0.11	2.29	0.02 – 0.21	0.022
Treatment (low amplification):Anti-expert sentiments	0.04	1.97	0.00 – 0.08	0.049
Treatment (high amplification):Anti-expert sentiments	-0.01	-0.33	-0.05 – 0.03	0.744
Treatment (correction with link):Anti-expert sentiments	0.02	0.93	-0.02 – 0.06	0.352
Random effects	*Variance*		mR^2	0.32
Participants (intercept)	0.74	0.86	cR^2	0.78
False News Posts (intercept)	0.01	0.08		
Residual	0.36	0.60		
**Sharing**				
Fixed effects	*B*	*t*	*95% CI*	*p*
(Intercept)	0.88	8.10	0.67 – 1.09	<0.001
Anti-expert sentiments	0.55	20.00	0.50 – 0.60	<0.001
Treatment (low amplification)	-0.19	-3.02	-0.31 – -0.07	0.003
Treatment (high amplification)	-0.07	-1.10	-0.19 – 0.05	0.273
Treatment (correction with link)	-0.15	-2.41	-0.27 – -0.03	0.016
Gender (male)	0.11	2.65	0.03 – 0.20	0.008
Age (25-34)	0.00	0.03	-0.16 – 0.16	0.974
Age (35-44)	-0.27	-3.40	-0.42 – -0.11	0.001
Age (45-54)	-0.74	-9.34	-0.89 – -0.58	<0.001
Age (55-64)	-0.92	-11.24	-1.08 – -0.76	<0.001
Age (65+)	-1.06	-13.69	-1.21 – -0.91	<0.001
Education (technical or vocational degree)	-0.02	-0.32	-0.12 – 0.09	0.747
Education (university degree)	0.13	2.54	0.03 – 0.23	0.011
Treatment (low amplification):Anti-expert sentiments	0.05	2.47	0.01 – 0.09	0.014
Treatment (high amplification):Anti-expert sentiments	0.01	0.47	-0.03 – 0.05	0.642
Treatment (correction with link):Anti-expert sentiments	0.03	1.73	-0.00 – 0.07	0.083
Random effects	*Variance*	*SD*	mR^2	0.33
Participants (intercept)	0.79	0.89	cR^2	0.80
False News Posts (intercept)	0.01	0.08		
Residual	0.33	0.58		

Supplementary Table 2. The moderating role of susceptibility to social influence ($RQ_{Social\ influence}$) in the case of false news

Accuracy				
Fixed effects	*B*	*t*	*95% CI*	*p*
(Intercept)	1.42	15.88	1.24 – 1.59	<0.001
SSI	0.32	18.54	0.29 – 0.35	<0.001

Treatment (low amplification)	-0.19	-4.56	-0.28 – -0.11	<0.001
Treatment (high amplification)	-0.22	-5.29	-0.31 – -0.14	<0.001
Treatment (correction with link)	-0.26	-6.20	-0.35 – -0.18	<0.001
Gender (male)	0.11	3.88	0.05 – 0.16	<0.001
Age (25-34)	0.04	0.74	-0.06 – 0.14	0.461
Age (35-44)	0.04	0.84	-0.06 – 0.14	0.401
Age (45-54)	-0.08	-1.55	-0.18 – 0.02	0.122
Age (55-64)	-0.16	-2.85	-0.27 – -0.05	0.004
Age (65+)	-0.13	-2.48	-0.24 – -0.03	0.013
Education (technical or vocational degree)	-0.02	-0.57	-0.09 – 0.05	0.570
Education (university degree)	0.02	0.52	-0.05 – 0.08	0.603
Treatment (low amplification):SSI	0.04	2.48	0.01 – 0.07	0.013
Treatment (high amplification):SSI	0.04	2.55	0.01 – 0.07	0.011
Treatment (correction with link):SSI	0.06	3.82	0.03 – 0.09	<0.001
Random effects	*Variance*	*SD*	mR^2	0.24
Participants (intercept)	0.28	0.53	cR^2	0.59
False News Posts (intercept)	0.04	0.19		
Residual	0.37	0.61		
Liking				
Fixed effects	*B*	*t*	*95% CI*	*p*
(Intercept)	0.59	6.41	0.41 – 0.77	<0.001
SSI	0.62	28.91	0.58 – 0.66	<0.001
Treatment (low amplification)	-0.06	-1.35	-0.14 – 0.03	0.176
Treatment (high amplification)	-0.06	-1.45	-0.14 – 0.02	0.146
Treatment (correction with link)	-0.04	-0.86	-0.12 – 0.05	0.392
Gender (male)	0.21	5.57	0.14 – 0.29	<0.001
Age (25-34)	0.07	0.90	-0.08 – 0.21	0.368
Age (35-44)	-0.01	-0.11	-0.14 – 0.13	0.911
Age (45-54)	-0.19	-2.62	-0.33 – -0.05	0.009
Age (55-64)	-0.25	-3.35	-0.40 – -0.11	0.001
Age (65+)	-0.24	-3.27	-0.39 – -0.10	0.001
Education (technical or vocational degree)	-0.03	-0.60	-0.12 – 0.06	0.552
Education (university degree)	0.07	1.56	-0.02 – 0.16	0.119
Treatment (low amplification):SSI	-0.00	-0.07	-0.03 – 0.03	0.947
Treatment (high amplification):SSI	-0.01	-0.43	-0.04 – 0.02	0.670
Treatment (correction with link):SSI	0.00	0.04	-0.03 – 0.03	0.971
Random effects	*Variance*	*SD*	mR^2	0.39
Participants (intercept)	0.60	0.77	cR^2	0.77
False News Posts (intercept)	0.01	0.08		
Residual	0.36	0.60		
Sharing				
Fixed effects	*B*	*t*	*95% CI*	*p*
(Intercept)	0.63	6.72	0.44 – 0.81	<0.001
SSI	-0.09	-2.12	-0.17 – -0.01	0.034
Treatment (low amplification)	-0.10	-2.33	-0.18 – -0.02	0.020
Treatment (high amplification)	-0.11	-2.66	-0.19 – -0.03	0.008
Treatment (correction with link)	0.60	27.47	0.56 – 0.64	<0.001
Gender (male)	0.24	6.07	0.16 – 0.31	<0.001

Age (25-34)	0.08	1.03	-0.07 – 0.22	0.301
Age (35-44)	-0.04	-0.59	-0.18 – 0.10	0.558
Age (45-54)	-0.26	-3.48	-0.40 – -0.11	0.001
Age (55-64)	-0.30	-3.82	-0.45 – -0.15	<0.001
Age (65+)	-0.31	-4.12	-0.46 – -0.17	<0.001
Education (technical or vocational degree)	-0.04	-0.92	-0.14 – 0.05	0.359
Education (university degree)	0.09	1.89	-0.00 – 0.18	0.059
Treatment (low amplification):SSI	0.02	1.26	-0.01 – 0.05	0.209
Treatment (high amplification):SSI	0.02	1.52	-0.01 – 0.05	0.128
Treatment (correction with link):SSI	0.03	1.70	-0.00 – 0.06	0.089
Random effects	*Variance*	*SD*	mR^2	0.40
Participants (intercept)	0.64	0.80	cR^2	0.79
False News Posts (intercept)	0.01	0.08		
Residual	0.34	0.58		

Supplementary Table 3. The moderating role of cognitive reflection ($RQ_{\text{Cognitive reflection}}$) in the case of false news

Accuracy				
Fixed effects	*B*	*t*	*95% CI*	*p*
(Intercept)	2.60	32.30	2.44 – 2.75	<0.001
CRT	-0.10	-6.59	-0.13 – -0.07	<0.001
Treatment (low amplification)	-0.06	-2.30	-0.12 – -0.01	0.022
Treatment (high amplification)	-0.12	-4.15	-0.17 – -0.06	<0.001
Treatment (correction with link)	-0.05	-1.66	-0.10 – 0.01	0.097
Gender (male)	0.12	3.92	0.06 – 0.18	<0.001
Age (25-34)	-0.00	-0.00	-0.12 – 0.12	1.000
Age (35-44)	-0.11	-1.91	-0.22 – 0.00	0.057
Age (45-54)	-0.50	-8.80	-0.61 – -0.39	<0.001
Age (55-64)	-0.64	-10.93	-0.76 – -0.53	<0.001
Age (65+)	-0.72	-12.96	-0.83 – -0.61	<0.001
Education (technical or vocational degree)	0.01	0.20	-0.07 – 0.08	0.840
Education (university degree)	0.06	1.71	-0.01 – 0.14	0.087
Treatment (low amplification):CRT	-0.02	-1.52	-0.05 – 0.01	0.130
Treatment (high amplification):CRT	-0.01	-0.45	-0.04 – 0.02	0.654
Treatment (correction with link):CRT	-0.05	-3.10	-0.08 – -0.02	0.002
Random effects	*Variance*	*SD*	mR^2	0.13
Participants (intercept)	0.38	0.62	cR^2	0.59
False News Posts (intercept)	0.03	0.19		
Residual	0.37	0.61		
Liking				
Fixed effects	*B*	*t*	*95% CI*	*p*
(Intercept)	2.84	34.54	2.68 – 3.01	<0.001
CRT	-0.04	-1.33	-0.09 – 0.02	0.184
Treatment (low amplification)	-0.10	-3.56	-0.15 – -0.04	<0.001
Treatment (high amplification)	-0.02	-0.62	-0.07 – 0.04	0.538
Treatment (correction with link)	-0.21	-10.27	-0.25 – -0.17	<0.001

Gender (male)	0.24	5.14	0.15 – 0.33	<0.001
Age (25-34)	-0.00	-0.03	-0.18 – 0.17	0.979
Age (35-44)	-0.28	-3.24	-0.44 – -0.11	0.001
Age (45-54)	-0.92	-10.98	-1.09 – -0.76	<0.001
Age (55-64)	-1.10	-12.68	-1.27 – -0.93	<0.001
Age (65+)	-1.27	-15.44	-1.43 – -1.11	<0.001
Education (technical or vocational degree)	0.02	0.36	-0.09 – 0.13	0.720
Education (university degree)	0.15	2.78	0.04 – 0.26	0.005
Treatment (low amplification):CRT	-0.01	-0.95	-0.04 – 0.02	0.341
Treatment (high amplification):CRT	0.02	1.08	-0.01 – 0.05	0.279
Treatment (correction with link):CRT	-0.01	-0.70	-0.04 – 0.02	0.487
Random effects	*Variance*	*SD*	mR^2	0.21
Participants (intercept)	0.92	0.96	cR^2	0.78
False News Posts (intercept)	0.01	0.08		
Residual	0.36	0.60		
Sharing				
Fixed effects	*B*	*t*	*95% CI*	*p*
(Intercept)	2.85	34.10	2.68 – 3.01	<0.001
CRT	-0.22	-10.76	-0.26 – -0.18	<0.001
Treatment (low amplification)	-0.07	-2.39	-0.12 – -0.01	0.017
Treatment (high amplification)	-0.04	-1.64	-0.10 – 0.01	0.101
Treatment (correction with link)	-0.05	-1.83	-0.10 – 0.00	0.068
Gender (male)	0.26	5.58	0.17 – 0.36	<0.001
Age (25-34)	0.01	0.09	-0.17 – 0.19	0.928
Age (35-44)	-0.31	-3.59	-0.48 – -0.14	<0.001
Age (45-54)	-0.99	-11.53	-1.16 – -0.82	<0.001
Age (55-64)	-1.14	-12.86	-1.32 – -0.97	<0.001
Age (65+)	-1.34	-15.93	-1.50 – -1.17	<0.001
Education (technical or vocational degree)	0.00	0.06	-0.11 – 0.12	0.952
Education (university degree)	0.17	3.04	0.06 – 0.28	0.002
Treatment (low amplification):CRT	0.02	1.28	-0.01 – 0.05	0.199
Treatment (high amplification):CRT	0.00	0.24	-0.02 – 0.03	0.812
Treatment (correction with link):CRT	0.00	0.25	-0.02 – 0.03	0.802
Random effects	*Variance*	*SD*	mR^2	0.23
Participants (intercept)	0.96	0.98	cR^2	0.80
False News Posts (intercept)	0.01	0.08		
Residual	0.33	0.58		

Supplementary Table 4. The moderating role of anti-expert sentiments ($RQ_{Anti-expert}$) in the case of true news

Accuracy				
Fixed effects	*B*	*t*	*95% CI*	*p*
(Intercept)	2.08	23.08	1.90 – 2.26	<0.001
Anti-expert sentiments	0.16	6.03	0.11 – 0.21	<0.001
Treatment (low amplification)	0.02	0.20	-0.18 – 0.22	0.845
Treatment (high amplification)	-0.10	-0.94	-0.30 – 0.10	0.348

Treatment (miscorrection with link)	-0.24	-2.35	-0.44 – -0.04	0.019
Gender (male)	0.06	2.15	0.01 – 0.12	0.031
Age (25-34)	0.06	1.16	-0.04 – 0.17	0.246
Age (35-44)	0.05	0.86	-0.06 – 0.15	0.388
Age (45-54)	-0.03	-0.65	-0.14 – 0.07	0.516
Age (55-64)	-0.07	-1.29	-0.17 – 0.04	0.197
Age (65+)	-0.03	-0.51	-0.13 – 0.07	0.608
Education (technical or vocational degree)	0.00	0.08	-0.07 – 0.07	0.940
Education (university degree)	0.12	3.65	0.06 – 0.19	<0.001
Treatment (low amplification):Anti-expert sentiments	-0.03	-0.76	-0.09 – 0.04	0.446
Treatment (high amplification):Anti-expert sentiments	-0.03	-0.77	-0.09 – 0.04	0.439
Treatment (miscorrection with link):Anti-expert sentiments	0.05	1.50	-0.02 – 0.12	0.134
Random effects	*Var.*	*SD*	*mR*^{2}	0.05
Participants (intercept)	0.20	0.45	*cR*^{2}	0.30
Residual	0.53	0.73		
Liking				
Fixed effects	*B*	*t*	*95% CI*	*p*
(Intercept)	1.03	8.19	0.78 – 1.27	<0.001
Anti-expert sentiments	0.54	15.48	0.47 – 0.61	<0.001
Treatment (low amplification)	0.08	0.63	-0.16 – 0.31	0.531
Treatment (high amplification)	0.04	0.31	-0.20 – 0.28	0.753
Treatment (miscorrection with link)	0.11	0.89	-0.13 – 0.34	0.371
Gender (male)	0.09	2.07	0.00 – 0.18	0.039
Age (25-34)	-0.04	-0.52	-0.21 – 0.12	0.601
Age (35-44)	-0.21	-2.57	-0.36 – -0.05	0.01
Age (45-54)	-0.58	-7.26	-0.74 – -0.43	<0.001
Age (55-64)	-0.71	-8.54	-0.87 – -0.55	<0.001
Age (65+)	-0.76	-9.65	-0.91 – -0.61	<0.001
Education (technical or vocational degree)	-0.01	-0.12	-0.11 – 0.10	0.904
Education (university degree)	0.09	1.79	-0.01 – 0.19	0.073
Treatment (low amplification):Anti-expert sentiments	-0.04	-0.96	-0.12 – 0.04	0.337
Treatment (high amplification):Anti-expert sentiments	-0.05	-1.37	-0.13 – 0.02	0.171
Treatment (miscorrection with link):Anti-expert sentiments	-0.04	-1.02	-0.12 – 0.04	0.308
Random effects	*Var.*		*mR*^{2}	0.22
Participants (intercept)	0.66	0.81	*cR*^{2}	0.60
Residual	0.69	0.83		
Sharing				
Fixed effects	*B*	*t*	*95% CI*	*p*
(Intercept)	0.84	7	0.60 – 1.08	<0.001
Anti-expert sentiments	0.58	17.97	0.52 – 0.65	<0.001
Treatment (low amplification)	0.10	0.99	-0.10 – 0.30	0.323
Treatment (high amplification)	0.06	0.58	-0.14 – 0.26	0.562

Treatment (amplification + link)	0.13	1.31	-0.07 – 0.33	0.190
Gender (male)	0.11	2.53	0.03 – 0.20	0.011
Age (25-34)	0.00	0.02	-0.17 – 0.17	0.985
Age (35-44)	-0.27	-3.24	-0.43 – -0.10	0.001
Age (45-54)	-0.70	-8.60	-0.86 – -0.54	<0.001
Age (55-64)	-0.82	-9.70	-0.99 – -0.65	<0.001
Age (65+)	-0.98	-12.21	-1.14 – -0.82	<0.001
Education (technical or vocational degree)	0.02	0.32	-0.09 – 0.12	0.746
Education (university degree)	0.16	3.11	0.06 – 0.27	0.002
Treatment (low amplification):Anti-expert sentiments	-0.05	-1.53	-0.11 – 0.01	0.126
Treatment (high amplification):Anti-expert sentiments	-0.06	-1.79	-0.12 – 0.01	0.073
Treatment (correction with link):Anti-expert sentiments	-0.06	-1.93	-0.13 – 0.00	0.054
Random effects	*Var.*		mR^2	0.28
Participants (intercept)	0.77	0.88	cR^2	0.73
Residual	0.46	0.68		

Supplementary Table 5. The moderating role of susceptibility to social influence ($RQ_{Social\ influence}$) in the case of true news

Accuracy				
Fixed effects	*B*	*t*	*95% CI*	*p*
(Intercept)	1.97	26.32	1.82 – 2.12	<0.001
SSI	0.18	8.39	0.14 – 0.22	<0.001
Treatment (low amplification)	-0.06	-0.84	-0.19 – 0.07	0.398
Treatment (high amplification)	-0.27	-3.95	-0.40 – -0.14	<0.001
Treatment (miscorrection with link)	-0.18	-2.69	-0.32 – -0.05	0.007
Gender (male)	0.09	3.39	0.04 – 0.15	0.001
Age (25-34)	0.09	1.60	-0.02 – 0.19	0.109
Age (35-44)	0.12	2.30	0.02 – 0.22	0.021
Age (45-54)	0.13	2.50	0.03 – 0.24	0.013
Age (55-64)	0.14	2.56	0.03 – 0.25	0.011
Age (65+)	0.23	4.18	0.12 – 0.33	<0.001
Education (technical or vocational degree)	-0.01	-0.18	-0.07 – 0.06	0.857
Education (university degree)	0.11	3.38	0.05 – 0.17	0.001
Treatment (low amplification):SSI	0.00	0.04	-0.05 – 0.05	0.970
Treatment (high amplification):SSI	0.04	1.65	-0.01 – 0.09	0.098
Treatment (miscorrection with link):SSI	0.04	1.42	-0.01 – 0.09	0.157
Random effects	*Var.*		mR^2	0.07
Participants (intercept)	0.18	0.42	cR^2	0.30
Residual	0.53	0.73		
Liking				
Fixed effects	*B*	*t*	*95% CI*	*p*
(Intercept)	0.64	6.31	0.44 – 0.83	<0.001
SSI	0.64	23.22	0.59 – 0.69	<0.001
Treatment (low amplification)	0.02	0.26	-0.13 – 0.18	0.796

Treatment (high amplification)	-0.13	-1.57	-0.28 – 0.03	0.118
Treatment (miscorrection with link)	0.06	0.77	-0.10 – 0.22	0.442
Gender (male)	0.20	5.07	0.12 – 0.28	<0.001
Age (25-34)	0.02	0.28	-0.13 – 0.17	0.78
Age (35-44)	0.02	0.23	-0.12 – 0.16	0.815
Age (45-54)	-0.07	-0.95	-0.22 – 0.08	0.343
Age (55-64)	-0.05	-0.69	-0.21 – 0.10	0.491
Age (65+)	0.03	0.36	-0.12 – 0.18	0.723
Education (technical or vocational degree)	-0.03	-0.72	-0.13 – 0.06	0.469
Education (university degree)	0.05	1.19	-0.04 – 0.15	0.234
Treatment (low amplification):SSI	-0.02	-0.82	-0.08 – 0.03	0.413
Treatment (high amplification):SSI	0.00	0.15	-0.06 – 0.06	0.877
Treatment (amplification + link):SSI	-0.04	-1.16	-0.10 – 0.02	0.245
Random effects	*Var.*		mR^2	0.31
Participants (intercept)	0.48	0.70	cR^2	0.60
Residual	0.69	0.83		
Sharing				
Fixed effects	*B*	*t*	*95% CI*	*p*
(Intercept)	0.47	4.83	0.28 – 0.66	<0.001
SSI	0.68	26.42	0.63 – 0.73	<0.001
Treatment (low amplification)	-0.01	-0.11	-0.14 – 0.12	0.911
Treatment (high amplification)	-0.08	-1.24	-0.22 – 0.05	0.214
Treatment (miscorrection with link)	0.01	0.12	-0.12 – 0.14	0.905
Gender (male)	0.23	5.80	0.15 – 0.31	<0.001
Age (25-34)	0.07	0.93	-0.08 – 0.22	0.351
Age (35-44)	-0.03	-0.40	-0.17 – 0.11	0.693
Age (45-54)	-0.16	-2.18	-0.31 – -0.02	0.029
Age (55-64)	-0.13	-1.65	-0.29 – 0.02	0.099
Age (65+)	-0.15	-1.94	-0.30 – 0.00	0.053
Education (technical or vocational degree)	-0.01	-0.27	-0.11 – 0.08	0.786
Education (university degree)	0.12	2.65	0.03 – 0.21	0.008
Treatment (low amplification):SSI	-0.02	-0.72	-0.07 – 0.03	0.474
Treatment (high amplification):SSI	-0.01	-0.43	-0.06 – 0.04	0.667
Treatment (miscorrection with link):SSI	-0.03	-1.18	-0.08 – 0.02	0.240
Random effects	*Var.*		mR^2	0.39
Participants (intercept)	0.57	0.75	cR^2	0.73
Residual	0.46	0.68		

Supplementary Table 6. The moderating role of cognitive reflection ($RQ_{Cognitive\ reflection}$) in the case of true news

Accuracy				
Fixed effects	*B*	*t*	*95% CI*	*p*
(Intercept)	2.62	48.06	2.51 – 2.73	<0.001
CRT	-0.05	-2.61	-0.09 – -0.01	0.009
Treatment (low amplification)	-0.06	-1.39	-0.15 – 0.03	0.165
Treatment (high amplification)	-0.15	-3.28	-0.24 – -0.06	0.001
Treatment (miscorrection with link)	-0.04	-0.93	-0.13 – 0.05	0.351

Gender (male)	0.10	3.46	0.04 – 0.16	0.001
Age (25-34)	0.07	1.20	-0.04 – 0.18	0.231
Age (35-44)	0.03	0.61	-0.07 – 0.14	0.540
Age (45-54)	-0.10	-1.94	-0.21 – 0.00	0.052
Age (55-64)	-0.13	-2.36	-0.24 – -0.02	0.018
Age (65+)	-0.10	-1.97	-0.20 – -0.00	0.049
Education (technical or vocational degree)	0.01	0.19	-0.06 – 0.08	0.852
Education (university degree)	0.13	3.93	0.07 – 0.20	<0.001
Treatment (low amplification):CRT	0.01	0.31	-0.04 – 0.05	0.758
Treatment (high amplification):CRT	-0.01	-0.61	-0.06 – 0.03	0.543
Treatment (miscorrection with link):CRT	-0.04	-1.59	-0.09 – 0.01	0.111
Random effects	*Var.*		mR^2	0.03
Participants (intercept)	0.21	0.46	cR^2	0.30
Residual	0.53	0.73		
Liking				
Fixed effects	*B*	*t*	*95% CI*	*p*
(Intercept)	2.91	34.72	2.74 – 3.07	<0.001
CRT	-0.19	-7.26	-0.24 – -0.14	<0.001
Treatment (low amplification)	-0.02	-0.41	-0.13 – 0.08	0.685
Treatment (high amplification)	-0.11	-2.02	-0.21 – -0.00	0.044
Treatment (miscorrection with link)	0.05	1.00	-0.05 – 0.16	0.320
Gender (male)	0.22	4.75	0.13 – 0.31	<0.001
Age (25-34)	-0.04	-0.49	-0.22 – 0.13	0.628
Age (35-44)	-0.25	-2.89	-0.42 – -0.08	0.004
Age (45-54)	-0.81	-9.49	-0.98 – -0.64	<0.001
Age (55-64)	-0.91	-10.31	-1.08 – -0.74	<0.001
Age (65+)	-1.01	-12.08	-1.17 – -0.84	<0.001
Education (technical or vocational degree)	0.01	0.17	-0.10 – 0.12	0.865
Education (university degree)	0.13	2.39	0.02 – 0.24	0.017
Treatment (low amplification):CRT	0.00	-0.09	-0.06 – 0.05	0.931
Treatment (high amplification):CRT	0.00	0.01	-0.06 – 0.06	0.988
Treatment (miscorrection with link):CRT	-0.05	-1.55	-0.10 – 0.01	0.122
Random effects	*Var.*	*SD*	mR^2	0.14
Participants (intercept)	0.79	0.89	cR^2	0.60
Residual	0.69	0.83		
Sharing				
Fixed effects	*B*	*t*	*95% CI*	*p*
(Intercept)	2.89	34.51	2.72 – 3.05	<0.001
CRT	-0.21	-8.68	-0.26 – -0.16	<0.001
Treatment (low amplification)	-0.03	-0.70	-0.12 – 0.06	0.483
Treatment (high amplification)	-0.13	-2.80	-0.21 – -0.04	0.005
Treatment (miscorrection with link)	-0.01	-0.33	-0.10 – 0.07	0.744
Gender (male)	0.26	5.32	0.16 – 0.35	<0.001
Age (25-34)	0.00	0.01	-0.18 – 0.18	0.990
Age (35-44)	-0.31	-3.52	-0.48 – -0.14	<0.001
Age (45-54)	-0.95	-10.82	-1.12 – -0.78	<0.001

Age (55-64)	-1.04	-11.46	-1.21 – -0.86	<0.001
Age (65+)	-1.24	-14.57	-1.41 – -1.08	<0.001
Education (technical or vocational degree)	0.04	0.61	-0.08 – 0.15	0.540
Education (university degree)	0.21	3.64	0.10 – 0.32	<0.001
Treatment (low amplification):CRT	-0.01	-0.29	-0.05 – 0.04	0.771
Treatment (high amplification):CRT	0.01	0.58	-0.03 – 0.06	0.559
Treatment (miscorrection with link):CRT	-0.03	-1.14	-0.08 – 0.02	0.253
Random effects	*Var.*	*SD*	mR^2	0.20
Participants (intercept)	0.91	0.96	cR^2	0.73
Residual	0.46	0.68		

Note. The mixed-effects regressions for false news for $RQ_{Anti\text{-}expert}$ were run on 9,842 observations (perceived accuracy), 9,846 observations (like), and 9,845 observations (share) with 1,926 respondents and 10 false news posts. The mixed-effects regressions for $RQ_{Social\ influence}$ were run on 9,842 observations (perceived accuracy), 9,846 observations (like), and 9,845 observations (share) with 1,926 respondents and 10 false news posts. The mixed-effects regressions for $RQ_{Cognitive\ reflection}$ were run on 9,848 observations (perceived accuracy), 9,852 observations (like), and 9,851 observations (share) with 1,927 respondents and 10 false news posts.
The mixed-effects regressions for true news for $RQ_{Anti\text{-}expert}$ were run on 5,805 observations (perceived accuracy), 5,803 observations (like), and 5,802 observations (share) with 1,926 respondents and 10 false news posts. The mixed-effects regressions for $RQ_{Social\ influence}$ were run on 5,805 observations (perceived accuracy), 5,803 observations (like), and 5,802 observations (share) with 1,926 respondents and 10 false news posts. The mixed-effects regressions for $RQ_{Cognitive\ reflection}$ were run on 5,808 observations (perceived accuracy), 5,806 observations (like), and 5,802 observations (share) with 1,927 respondents and 10 false news posts.
As we showed the same three true news posts to all our respondents, we simplified the structure of our mixed-effects regressions by removing the random intercept for social media posts. That is, we computed the following model: response ~ social miscorrection treatment:individual differences + gender + age + education + (1|respondent id).
More detailed analyses (including our code and data) can be found on OSF (https://osf.io/4hjcf/?view_only=17b4b2f95f5c465e900b30046c0210b6, files analysis_fakenews_osf and analysis_truenews_osf).

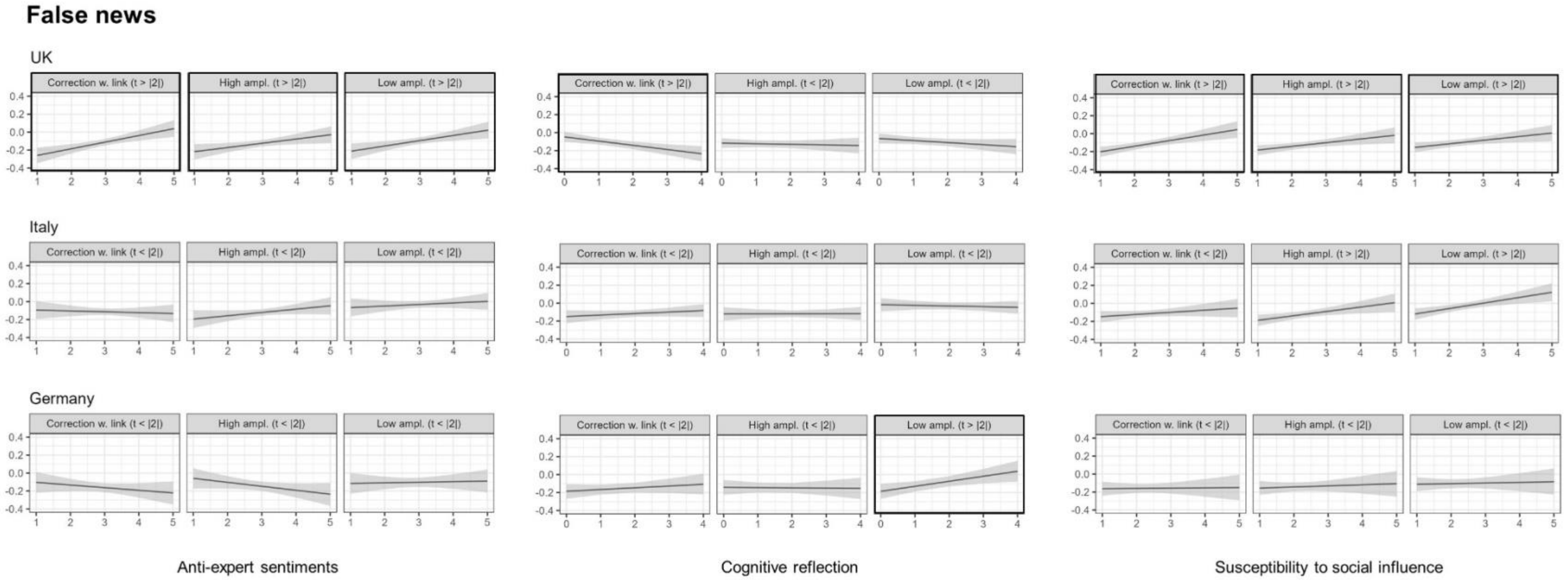

Supplementary Figure 1. Marginal effects plots for the effect of social corrections on perceived accuracy for anti-expert sentiments (left), cognitive reflection capacities (middle), and susceptibility to social influence (right)—for all three countries and for false news. Bold frames indicate significant interactions. We test linear interaction effect (LIE) assumption as suggested by Hainmueller, Mummolo, and Xu (2018). Our data does not fulfill this assumption (see markdownfiles on our OSF repositories). We computed the same analyses for “liking” and sharing posts. We did not find that any of the suggested individual differences moderate the effect of social corrections on the probability of “liking” and sharing posts. Overall, the effect of social corrections is not moderated by anti-expert sentiments, cognitive reflection capacities, or susceptibility to social influence.

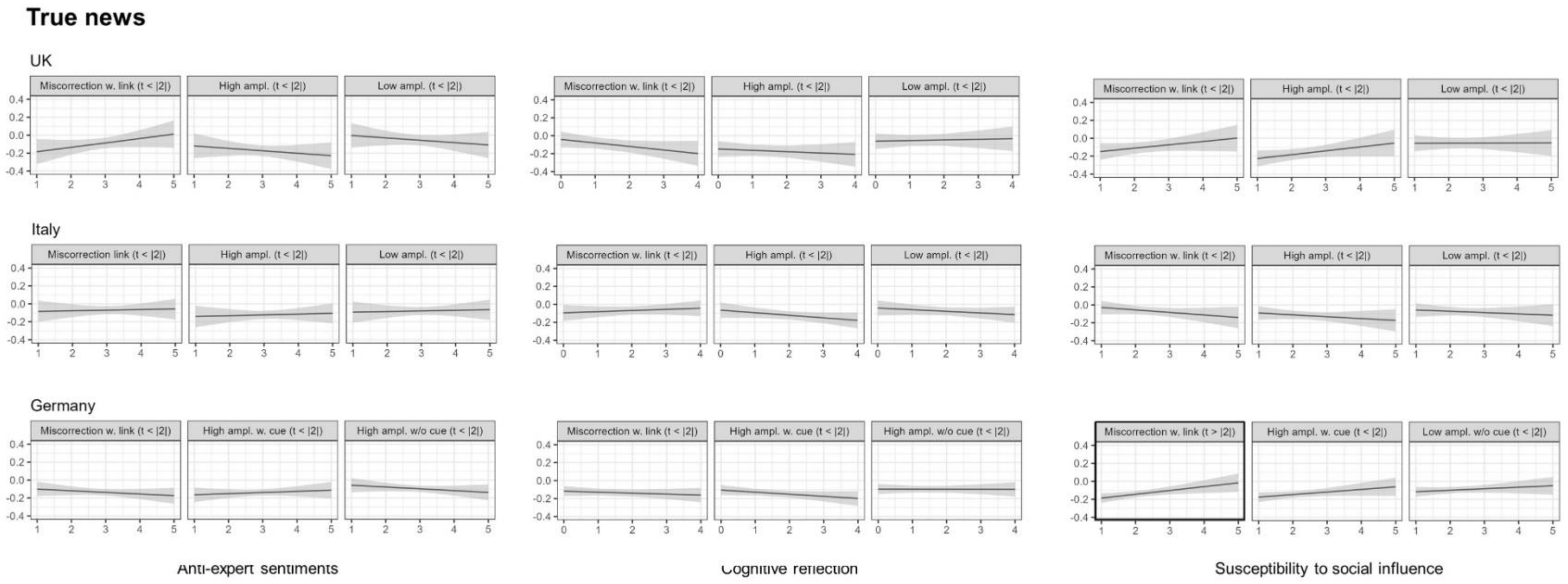

Supplementary Figure 2. Marginal effects plots for the effect of social corrections on perceived accuracy for anti-expert sentiments (left), cognitive reflection capacities (middle), and susceptibility to social influence (right)—for all three countries and for true news. Bold frames indicate significant interactions. We test linear interaction effect (LIE) assumption as suggested by Hainmueller, Mummolo, and Xu (2018). Our data does not fulfill this assumption (see markdownfiles on our OSF repositories). We computed the same analyses for “liking” and sharing posts. We did not find that any of the suggested individual differences moderate the effect of social corrections on the probability of “liking” and sharing posts. Overall, the effect of social corrections is not moderated by anti-expert sentiments, cognitive reflection capacities, or susceptibility to social influence

Supplementary Table 7. Results of linear mixed-effects models for $H_{Correct\ false}$ in the UK excluding respondents that failed attention checks.

**Perceived accuracy**				
Fixed effects	*B*	*t*	*95% CI*	*p*
(Intercept)	2.40	29.88	2.24 – 2.55	<0.001
Treatment (low amplification)	-0.10	-5.21	-0.14 – -0.06	<0.001
Treatment (high amplification)	-0.13	-6.73	-0.17 – -0.09	<0.001
Treatment (correction with link)	-0.12	-6.16	-0.16 – -0.08	<0.001
Gender (male)	0.09	2.71	0.02 – 0.15	0.007
Age (25-34)	0.03	0.40	-0.10 – 0.15	0.686
Age (35-44)	-0.08	-1.32	-0.19 – 0.04	0.188
Age (45-54)	-0.44	-7.52	-0.55 – -0.32	<0.001
Age (55-64)	-0.58	-9.70	-0.70 – -0.46	<0.001
Age (65+)	-0.64	-11.25	-0.75 – -0.53	<0.001
Education (technical or vocational degree)	0.02	0.62	-0.05 – 0.10	0.535
Education (university degree)	0.03	0.84	-0.04 – 0.10	0.404
Random effects	*Variance*	*SD*	mR^2	0.90
Participants (intercept)	0.37	0.61	cR^2	0.56
False News Posts (intercept)	0.04	0.20		
Residual	0.37	0.61		
**Liking**				
Fixed effects	*B*	*t*	*95% CI*	*p*
(Intercept)	2.46	30.73	2.30 – 2.62	<0.001
Treatment (low amplification)	-0.06	-3.32	-0.10 – -0.03	0.001
Treatment (high amplification)	-0.08	-4.22	-0.12 – -0.04	<0.001
Treatment (correction with link)	-0.04	-1.97	-0.08 – -0.00	0.048
Gender (male)	0.19	3.94	0.09 – 0.28	<0.001
Age (25-34)	0.04	0.40	-0.15 – 0.22	0.688
Age (35-44)	-0.24	-2.71	-0.41 – -0.07	0.007
Age (45-54)	-0.84	-9.61	-1.01 – -0.66	<0.001
Age (55-64)	-1.02	-11.43	-1.19 – -0.84	<0.001
Age (65+)	-1.15	-13.64	-1.32 – -0.99	<0.001
Education (technical or vocational degree)	0.04	0.77	-0.07 – 0.16	0.441
Education (university degree)	0.10	1.84	-0.01 – 0.21	0.066
Random effects	*Variance*	*SD*	mR^2	0.15
Participants (intercept)	0.91	0.95	cR^2	0.76
False News Posts (intercept)	0.01	0.10		
Residual	0.36	0.60		
**Sharing**				
Fixed effects	*B*	*t*	*95% CI*	*p*
(Intercept)	2.43	29.80	2.27 – 2.59	<0.001
Treatment (low amplification)	-0.04	-2.05	-0.07 – -0.00	0.040
Treatment (high amplification)	-0.05	-2.61	-0.08 – -0.01	0.009
Treatment (correction with link)	-0.04	-2.34	-0.08 – -0.01	0.019
Gender (male)	0.21	4.33	0.11 – 0.30	<0.001
Age (35-44)	0.05	0.52	-0.14 – 0.24	0.605
Age (45-54)	-0.26	-2.86	-0.43 – -0.08	0.004
Age (55-64)	-0.89	-10.04	-1.06 – -0.72	<0.001
Age (65-75)	-1.04	-11.47	-1.22 – -0.86	<0.001

Age (76+)	-1.20	-13.96	-1.37 – -1.03	<0.001
Education (technical or vocational degree)	0.03	0.50	-0.09 – 0.14	0.618
Education (university degree)	0.12	2.13	0.01 – 0.23	0.033
Random effects	*Variance*	*SD*	*mR*^{2}	0.16
Participants (intercept)	0.95	0.97	*cR*^{2}	0.79
False News Posts (intercept)	0.01	0.10		
Residual	0.33	0.57		

Note. The mixed-effects regressions were run on 9,331 observations (perceived accuracy), 9,335 observations (like), and 9,335 observations (share) with 9,840 respondents and 10 false news posts. *mR*^{2} = marginal R^2 (i.e., variance of the fixed effects); *cR*^{2} = conditional R^2 (i.e., variance of the fixed and random effects). Reference groups: Treatment = control condition; gender = female; age = 18-24; education = less than primary education.

Supplementary Table 8. Results of linear mixed-effects models for $H_{\text{Correct false}}$ in the UK controlling for congeniality.

**Perceived accuracy**				
Fixed effects	*B*	*t*	*95% CI*	*p*
(Intercept)	2.45	31.43	2.29 – 2.60	<0.001
Treatment (low amplification)	-0.10	-5.10	-0.13 – -0.06	<0.001
Treatment (high amplification)	-0.13	-6.73	-0.16 – -0.09	<0.001
Treatment (correction with link)	-0.11	-6.05	-0.15 – -0.08	<0.001
Gender (male)	0.10	3.21	0.04 – 0.16	0.001
Age (35-44)	0.04	0.73	-0.08 – 0.17	0.463
Age (45-54)	-0.05	-0.91	-0.17 – 0.06	0.364
Age (55-64)	-0.47	-8.06	-0.58 – -0.35	<0.001
Age (65-75)	-0.63	-10.52	-0.75 – -0.52	<0.001
Age (76+)	-0.70	-12.31	-0.81 – -0.59	<0.001
Education (technical or vocational degree)	0.00	0.08	-0.07 – 0.08	0.935
Education (university degree)	0.02	0.52	-0.05 – 0.09	0.605
Congeniality (congenial)	0.02	0.99	-0.02 – 0.05	0.322
Random effects	*Variance*	*SD*	*mR*^{2}	0.11
Participants (intercept)	0.40	0.63	*cR*^{2}	0.59
False News Posts (intercept)	0.03	0.17		
Residual	0.37	0.61		
**Liking**				
Fixed effects	*B*	*t*	*95% CI*	*p*
(Intercept)	2.54	32.02	2.38 – 2.69	<0.001
Treatment (low amplification)	-0.06	-3.02	-0.09 – -0.02	0.003
Treatment (high amplification)	-0.08	-4.17	-0.12 – -0.04	<0.001
Treatment (correction with link)	-0.03	-1.77	-0.07 – 0.00	0.078
Gender (male)	0.20	4.25	0.11 – 0.30	<0.001
Age (35-44)	0.08	0.82	-0.10 – 0.25	0.412
Age (45-54)	-0.18	-2.00	-0.35 – -0.00	0.045
Age (55-64)	-0.86	-9.94	-1.04 – -0.69	<0.001
Age (65-75)	-1.09	-12.07	-1.26 – -0.91	<0.001
Age (76+)	-1.23	-14.50	-1.40 – -1.07	<0.001
Education (technical or vocational degree)	0.01	0.23	-0.10 – 0.13	0.821
Education (university degree)	0.08	1.37	-0.03 – 0.19	0.172

Congeniality (congenial)	0.02	1.36	-0.01 – 0.05	0.175
Random effects	*Variance*	*SD*	*mR²*	0.17
Participants (intercept)	0.99	0.99	*cR²*	0.78
False News Posts (intercept)	0.01	0.32		
Residual	0.36	0.60		
**Sharing**				
Fixed effects	*B*	*t*	*95% CI*	*p*
(Intercept)	2.51	31.35	2.36 – 2.67	<0.001
Treatment (low amplification)	-0.04	-2.09	-0.07 – -0.00	0.037
Treatment (high amplification)	-0.04	-2.24	-0.08 – -0.01	0.025
Treatment (correction with link)	-0.05	-2.48	-0.08 – -0.01	0.013
Gender (male)	0.23	4.72	0.13 – 0.32	<0.001
Age (35-44)	0.09	0.91	-0.10 – 0.27	0.360
Age (45-54)	-0.21	-2.35	-0.39 – -0.03	0.019
Age (55-64)	-0.93	-10.49	-1.10 – -0.76	<0.001
Age (65-75)	-1.13	-12.26	-1.30 – -0.95	<0.001
Age (76+)	-1.30	-14.98	-1.47 – -1.13	<0.001
Education (technical or vocational degree)	-0.00	-0.05	-0.12 – 0.11	0.964
Education (university degree)	0.09	1.65	-0.02 – 0.21	0.099
Congeniality (congenial)	0.03	2.15	0.00 – 0.06	0.032
Random effects	*Variance*	*SD*	*mR²*	0.18
Participants (intercept)	1.03	1.01	*cR²*	0.80
False News Posts (intercept)	0.01	0.10		
Residual	0.33	0.57		

Note. The mixed-effects regressions were run on 9,841 observations (perceived accuracy), 9,840 observations (like), and 9,840 observations (share) with 9,840 respondents and 10 false news posts. mR^2 = marginal R^2 (i.e., variance of the fixed effects); cR^2 = conditional R^2 (i.e., variance of the fixed and random effects). Reference groups: Treatment = control condition; gender = female; age = 18-24; education = less than primary education; congeniality = not congenial.

Supplementary Table 9. Results of linear mixed-effects models for $H_{\text{Miscorrect true}}$ in the UK excluding respondents that failed attention checks.

**Perceived accuracy**				
Fixed effects	*B*	*t*	*95% CI*	*p*
(Intercept)	2.52	50.61	2.42 – 2.62	<0.001
Treatment (low amplification)	-0.06	-1.87	-0.12 – 0.00	0.061
Treatment (high amplification)	-0.18	-5.68	-0.24 – -0.12	<0.001
Treatment (correction with link)	-0.10	-3.37	-0.17 – -0.04	0.001
Gender (male)	0.08	2.67	0.02 – 0.14	0.008
Age (35-44)	0.08	1.31	-0.04 – 0.19	0.190
Age (45-54)	0.03	0.46	-0.08 – 0.13	0.647
Age (55-64)	-0.07	-1.31	-0.18 – 0.04	0.192
Age (65-75)	-0.10	-1.80	-0.21 – 0.01	0.072
Age (76+)	-0.06	-1.14	-0.16 – 0.04	0.255
Education (technical or vocational degree)	0.02	0.54	-0.05 – 0.09	0.587
Education (university degree)	0.12	3.64	0.06 – 0.19	<0.001
Random effects	*Variance*	*SD*	*mR²*	0.02

Participants (intercept)	0.20	0.45	cR^2	0.28
Residual	0.54	0.73		
**Liking**				
Fixed effects	*B*	*t*	*95% CI*	*p*
(Intercept)	2.56	32.27	2.40 – 2.71	<0.001
Treatment (low amplification)	-0.04	-1.10	-0.11 – 0.03	0.274
Treatment (high amplification)	-0.12	-3.28	-0.19 – -0.05	0.001
Treatment (correction with link)	-0.02	-0.55	-0.09 – 0.05	0.584
Gender (male)	0.17	3.51	0.07 – 0.26	<0.001
Age (35-44)	0.01	0.07	-0.18 – 0.19	0.941
Age (45-54)	-0.20	-2.19	-0.37 – -0.02	0.029
Age (55-64)	-0.72	-8.09	-0.89 – -0.54	<0.001
Age (65-75)	-0.82	-9.07	-1.00 – -0.65	<0.001
Age (76+)	-0.89	-10.32	-1.06 – -0.72	<0.001
Education (technical or vocational degree)	0.03	0.43	-0.09 – 0.14	0.670
Education (university degree)	0.08	1.39	-0.03 – 0.19	0.165
Random effects	*Variance*	*SD*	mR^2	0.09
Participants (intercept)	0.78	0.88	cR^2	0.57
Residual	0.70	0.84		
**Sharing**				
Fixed effects	*B*	*t*	*95% CI*	*p*
(Intercept)	2.48	31.07	2.33 – 2.64	<0.001
Treatment (low amplification)	-0.05	-1.52	-0.11 – 0.01	0.128
Treatment (high amplification)	-0.11	-3.66	-0.17 – -0.05	<0.001
Treatment (correction with link)	-0.05	-1.74	-0.11 – 0.01	0.082
Gender (male)	0.20	4.02	0.10 – 0.29	<0.001
Age (35-44)	0.05	0.54	-0.14 – 0.24	0.589
Age (45-54)	-0.25	-2.71	-0.43 – -0.07	0.007
Age (55-64)	-0.84	-9.32	-1.02 – -0.67	<0.001
Age (65-75)	-0.93	-10.04	-1.11 – -0.75	<0.001
Age (76+)	-1.11	-12.58	-1.28 – -0.93	<0.001
Education (technical or vocational degree)	0.06	0.95	-0.06 – 0.17	0.343
Education (university degree)	0.15	2.61	0.04 – 0.26	0.009
Random effects	*Variance*	*SD*	mR^2	0.14
Participants (intercept)	0.91	0.95	cR^2	0.71
Residual	0.46	0.68		

Note. The mixed-effects regressions were run on 5,508 observations (perceived accuracy), 5,506 observations (like), and 5,505 observations (share) with 9,840 respondents and 3 false news posts. mR^2 = marginal R^2 (i.e., variance of the fixed effects); cR^2 = conditional R^2 (i.e., variance of the fixed and random effects). Reference groups: Treatment = control condition; gender = female; age = 18-24; education = less than primary education.

Supplementary Table 10. Results of linear mixed-effects models for $H_{\text{Miscorrect true}}$ in the UK controlling for congeniality.

**Perceived accuracy**				
Fixed effects	*B*	*t*	*95% CI*	*p*
(Intercept)	2.49	49.56	2.39 – 2.59	<0.001
Treatment (low amplification)	-0.05	-1.81	-0.11 – 0.00	0.071

Treatment (high amplification)	-0.17	-5.81	-0.23 – -0.11	<0.001
Treatment (correction with link)	-0.10	-3.24	-0.16 – -0.04	0.001
Gender (male)	0.09	3.13	0.03 – 0.15	0.002
Age (35-44)	0.09	1.56	-0.02 – 0.20	0.118
Age (45-54)	0.06	1.10	-0.05 – 0.17	0.271
Age (55-64)	-0.08	-1.46	-0.18 – 0.03	0.145
Age (65-75)	-0.11	-2.00	-0.22 – -0.00	0.045
Age (76+)	-0.08	-1.44	-0.18 – 0.03	0.149
Education (technical or vocational degree)	0.01	0.15	-0.07 – 0.08	0.878
Education (university degree)	0.13	3.62	0.06 – 0.19	<0.001
Congeniality (congenial)	0.17	6.63	0.12 – 0.22	<0.001
Random effects	*Variance*	*SD*	mR^2	0.03
Participants (intercept)	0.24	0.49	cR^2	0.52
Residual	0.52	0.72		
**Liking**				
Fixed effects	*B*	*t*	*95% CI*	*p*
(Intercept)	2.51	31.16	2.35 – 2.67	<0.001
Treatment (low amplification)	-0.03	-0.79	-0.10 – 0.04	0.431
Treatment (high amplification)	-0.11	-3.19	-0.18 – -0.04	0.001
Treatment (correction with link)	-0.01	-0.21	-0.08 – 0.06	0.832
Gender (male)	0.20	3.96	0.10 – 0.29	<0.001
Age (35-44)	0.03	0.32	-0.16 – 0.22	0.752
Age (45-54)	-0.16	-1.74	-0.34 – 0.02	0.082
Age (55-64)	-0.74	-8.22	-0.92 – -0.56	<0.001
Age (65-75)	-0.87	-9.33	-1.05 – -0.69	<0.001
Age (76+)	-0.94	-10.70	-1.12 – -0.77	<0.001
Education (technical or vocational degree)	0.00	0.07	-0.11 – 0.12	0.945
Education (university degree)	0.09	1.51	-0.03 – 0.20	0.131
Congeniality (congenial)	0.33	10.64	0.27 – 0.39	<0.001
Random effects	*Variance*	*SD*	mR^2	0.12
Participants (intercept)	0.65	0.81	cR^2	0.63
Residual	0.92	0.96		
**Sharing**				
Fixed effects	*B*	*t*	*95% CI*	*p*
(Intercept)	2.54	31.63	2.38 – 2.69	<0.001
Treatment (low amplification)	-0.04	-1.50	-0.10 – 0.01	0.133
Treatment (high amplification)	-0.11	-3.69	-0.17 – -0.05	<0.001
Treatment (correction with link)	-0.05	-1.80	-0.11 – 0.00	0.072
Gender (male)	0.22	4.49	0.13 – 0.32	<0.001
Age (35-44)	0.08	0.85	-0.11 – 0.27	0.394
Age (45-54)	-0.21	-2.28	-0.39 – -0.03	0.022
Age (55-64)	-0.88	-9.69	-1.06 – -0.70	<0.001
Age (65-75)	-1.01	-10.73	-1.19 – -0.82	<0.001
Age (76+)	-1.20	-13.46	-1.37 – -1.02	<0.001
Education (technical or vocational degree)	0.03	0.50	-0.09 – 0.15	0.620
Education (university degree)	0.14	2.37	0.02 – 0.25	0.018
Congeniality (congenial)	0.13	4.76	0.07 – 0.18	<0.001
Random effects	*Variance*	*SD*	mR^2	0.16
Participants (intercept)	1.00	1.00	cR^2	0.74
Residual	0.45	0.67		

Note. The mixed-effects regressions were run on 5,808 observations (perceived accuracy), 5,806 observations (like), and 5,805 observations (share) with 9,840 respondents and 3 false news posts. mR^2 = marginal R^2 (i.e., variance of the fixed effects); cR^2 = conditional R^2 (i.e., variance of the fixed and random effects). Reference groups: Treatment = control condition; gender = female; age = 18-24; education = less than primary education.

Supplementary Table 11. Overview of demographics of UK Experiment.

**Demographic Variable**	**Frequency**	**Percentage (%)**
Gender		
Female	990	50.9
Male	946	48.7
Non-binary / third gender / other	8	0.4
Age		
18-24	235	12.1
25-34	285	14.7
35-44	344	17.7
45-54	355	18.3
55-64	310	15.9
65+	415	21.3
Education		
Low	771	39.7
Mid	530	27.3
High	643	33.1
Party		
Conservative	594	30.6
Labour	520	26.7
Liberal democrat	141	7.3
No PID	671	34.5
Other party	18	0.9

Note. Education: low = incomplete Secondary Education (Below GCSE / O Level), Secondary Education Completed (GCSE / O Level / CSE or equivalent), or Secondary Education Completed (A Level or equivalent); mid = some Vocational or Technical Qualifications or Vocational or Technical Qualifications Completed (e.g., HND, NVQ); high = University Education Completed (First Degree e.g., BA, BSc), Postgraduate Education Completed (e.g., Masters), Doctorate, or Post-doctorate or equivalent. Party: No PID = *no party* and *don't know* option.

Supplementary Methods

Italy Experiment (Experiment 2)

The following table gives an overview of the detailed results of the interaction of social corrections with anti-expert sentiments ($RQ_{Anti\text{-}expert}$), susceptibility to social influence ($RQ_{Social\ influence}$), and cognitive reflection ($RQ_{Cognitive\ reflection}$).

Supplementary Table 12. Results of linear mixed-effects models. The moderating role of anti-expert sentiments ($RQ_{Anti\text{-}expert}$) in the case of false news

**Accuracy**				
Fixed effects	*B*	*t*	*95% CI*	*p*
(Intercept)	1.21	13.91	1.04 – 1.38	<0.001
Treatment (low amplification)	-0.08	-1.15	-0.23 – 0.06	0.249
Treatment (high amplification)	-0.23	-3.15	-0.37 – -0.09	0.002
Treatment (correction with link)	-0.08	-1.15	-0.23 – 0.06	0.249
Anti-expert sentiments	0.28	13.17	0.24 – 0.32	<0.001
Gender (male)	0.00	-0.02	-0.05 – 0.05	0.986
Age (35-44)	-0.04	-0.9	-0.14 – 0.05	0.367
Age (45-54)	-0.16	-3.39	-0.25 – -0.07	0.001
Age (55-64)	-0.13	-2.71	-0.22 – -0.04	0.007
Age (65-75)	-0.18	-3.66	-0.28 – -0.08	<0.001
Age (76+)	-0.34	-1.71	-0.73 – 0.05	0.087
Education (technical or vocational degree)	-0.05	-1.41	-0.13 – 0.02	0.158
Education (university degree)	-0.09	-2.75	-0.15 – -0.03	0.006
Treatment (low amplification):Anti-expert sentiments	0.02	0.74	-0.03 – 0.06	0.461
Treatment (high amplification):Anti-expert sentiments	0.04	1.56	-0.01 – 0.08	0.118
Treatment (correction with link):Anti-expert sentiments	-0.01	-0.41	-0.06 – 0.04	0.679
Random effects	*Var.*		mR^2	0.08
Participants (intercept)	0.37	0.57	cR^2	0.52
False News Posts (intercept)	0.01	0.10		
Residual	0.37	0.61		
**Liking**				
Fixed effects	*B*	*t*	*95% CI*	*p*
(Intercept)	0.97	8.94	0.75 – 1.18	<0.001
Treatment (low amplification)	-0.13	-1.47	-0.31 – 0.04	0.140
Treatment (high amplification)	-0.16	-1.77	-0.33 – 0.02	0.076
Treatment (correction with link)	-0.11	-1.22	-0.29 – 0.07	0.221
Anti-expert sentiments	0.41	14.49	0.35 – 0.46	<0.001
Gender (male)	-0.06	-1.68	-0.13 – 0.01	0.092

Age (35-44)	-0.08	-1.21	-0.21 – 0.05	0.227
Age (45-54)	-0.17	-2.66	-0.29 – -0.04	0.008
Age (55-64)	-0.18	-2.86	-0.31 – -0.06	0.004
Age (65-75)	-0.31	-4.54	-0.44 – -0.17	<0.001
Age (76+)	-0.43	-1.57	-0.96 – 0.11	0.117
Education (technical or vocational degree)	-0.07	-1.38	-0.17 – 0.03	0.166
Education (university degree)	-0.09	-1.92	-0.17 – 0.00	0.055
Treatment (low amplification):Anti-expert sentiments	0.03	1.07	-0.03 – 0.09	0.285
Treatment (high amplification):Anti-expert sentiments	0.01	0.34	-0.05 – 0.07	0.735
Treatment (correction with link):Anti-expert sentiments	-0.01	-0.32	-0.07 – 0.05	0.751
Random effects	*Var.*		mR^2	0.10
Participants (intercept)	0.64	0.80	cR^2	0.59
False News Posts (intercept)	0.01	0.10		
Residual	0.54	0.73		
**Sharing**				
Fixed effects	*B*	*t*	*95% CI*	*p*
(Intercept)	0.82	7.44	0.60 – 1.03	<0.001
Treatment (low amplification)	0.03	0.4	-0.13 – 0.20	0.687
Treatment (high amplification)	-0.07	-0.8	-0.24 – 0.10	0.422
Treatment (correction with link)	0.05	0.52	-0.13 – 0.22	0.605
Anti-expert sentiments	0.44	15.45	0.38 – 0.49	<0.001
Gender (male)	-0.07	-2	-0.15 – -0.00	0.045
Age (35-44)	-0.08	-1.15	-0.22 – 0.06	0.249
Age (45-54)	-0.17	-2.59	-0.30 – -0.04	0.010
Age (55-64)	-0.18	-2.68	-0.31 – -0.05	0.007
Age (65-75)	-0.29	-4.13	-0.43 – -0.15	<0.001
Age (76+)	-0.38	-1.37	-0.94 – 0.17	0.171
Education (technical or vocational degree)	-0.03	-0.69	-0.12 – 0.06	0.491
Education (university degree)	-0.05	-0.87	-0.15 – 0.06	0.387
Treatment (low amplification):Anti-expert sentiments	-0.02	-0.84	-0.08 – 0.03	0.401
Treatment (high amplification):Anti-expert sentiments	-0.02	-0.7	-0.07 – 0.03	0.484
Treatment (correction with link):Anti-expert sentiments	-0.05	-1.93	-0.11 – 0.00	0.054
Random effects	*Var.*		mR^2	0.10
Participants (intercept)	0.70	0.84	cR^2	0.62
False News Posts (intercept)	0.01	0.10		
Residual	0.50	0.71		

Supplementary Table 13. Results of linear mixed-effects models. The moderating role of susceptibility to social influence ($RQ_{Social\ influence}$) in the case of false news

**Accuracy**				
Fixed effects	*B*	*t*	*95% CI*	*p*
(Intercept)	1.24	17.03	1.10 – 1.38	<0.001
Treatment (low amplification)	-0.18	-3.63	-0.27 – -0.08	<0.001
Treatment (high amplification)	-0.24	-4.82	-0.33 – -0.14	<0.001
Treatment (correction with link)	-0.17	-3.47	-0.27 – -0.07	0.001
SSI	0.29	16.88	0.25 – 0.32	<0.001
Gender (male)	0.01	0.31	-0.04 – 0.06	0.760
Age (35-44)	0.06	1.32	-0.03 – 0.15	0.186
Age (45-54)	-0.01	-0.19	-0.09 – 0.08	0.851
Age (55-64)	0.05	1.21	-0.03 – 0.14	0.227
Age (65-75)	0.05	1.05	-0.04 – 0.14	0.293
Age (76+)	-0.17	-0.91	-0.54 – 0.20	0.362
Education (technical or vocational degree)	-0.12	-3.8	-0.18 – -0.06	<0.001
Education (university degree)	-0.06	-1.77	-0.13 – 0.01	0.078
Treatment (low amplification):SSI	0.06	3.21	0.02 – 0.10	0.001
Treatment (high amplification):SSI	0.05	2.6	0.01 – 0.09	0.009
Treatment (correction with link):SSI	0.02	1.23	-0.01 – 0.06	0.218
Random effects	*Var.*		mR^2	0.14
Participants (intercept)	0.28	0.53	cR^2	0.52
False News Posts (intercept)	0.01	0.10		
Residual	0.37	0.61		

**Liking**				
Fixed effects	*B*	*t*	*95% CI*	*p*
(Intercept)	0.93	10.77	0.76 – 1.10	<0.001
Treatment (low amplification)	-0.23	-3.76	-0.34 – -0.11	<0.001
Treatment (high amplification)	-0.22	-3.69	-0.34 – -0.10	<0.001
Treatment (correction with link)	-0.22	-3.7	-0.34 – -0.10	<0.001
SSI	0.44	20.01	0.40 – 0.48	<0.001
Gender (male)	-0.05	-1.43	-0.11 – 0.02	0.154
Age (35-44)	0.07	1.17	-0.05 – 0.20	0.244
Age (45-54)	0.05	0.84	-0.07 – 0.17	0.399
Age (55-64)	0.08	1.36	-0.04 – 0.20	0.174
Age (65-75)	0.03	0.5	-0.09 – 0.16	0.617
Age (76+)	-0.18	-0.72	-0.68 – 0.32	0.474
Education (technical or vocational degree)	-0.13	-3.04	-0.21 – -0.04	0.002
Education (university degree)	-0.08	-1.76	-0.18 – 0.01	0.078
Treatment (low amplification):SSI	0.08	3.35	0.03 – 0.12	0.001
Treatment (high amplification):SSI	0.04	1.68	-0.01 – 0.08	0.093
Treatment (correction with link):SSI	0.04	1.54	-0.01 – 0.08	0.124
Random effects	*Var.*		mR^2	0.18
Participants (intercept)	0.54	0.73	cR^2	0.59
False News Posts (intercept)	0.01	0.10		

Residual	0.54	0.73		
**Sharing**				
Fixed effects	*B*	*t*	*95% CI*	*p*
(Intercept)	0.81	9.11	0.63 – 0.98	<0.001
Treatment (low amplification)	-0.21	-3.64	-0.32 – -0.10	<0.001
Treatment (high amplification)	-0.22	-3.75	-0.33 – -0.10	<0.001
Treatment (correction with link)	-0.13	-2.21	-0.24 – -0.01	0.027
SSI	0.47	21.3	0.42 – 0.51	<0.001
Gender (male)	-0.06	-1.77	-0.13 – 0.01	0.077
Age (35-44)	0.08	1.2	-0.05 – 0.20	0.229
Age (45-54)	0.05	0.89	-0.06 – 0.17	0.373
Age (55-64)	0.09	1.52	-0.03 – 0.21	0.130
Age (65-75)	0.06	0.95	-0.07 – 0.19	0.342
Age (76+)	-0.13	-0.51	-0.64 – 0.38	0.613
Education (technical or vocational degree)	-0.07	-1.68	-0.15 – 0.01	0.093
Education (university degree)	-0.06	-1.22	-0.15 – 0.04	0.224
Treatment (low amplification):SSI	0.07	3.25	0.03 – 0.12	0.001
Treatment (high amplification):SSI	0.04	1.7	-0.01 – 0.08	0.089
Treatment (correction with link):SSI	0	0.22	-0.04 – 0.05	0.827
Random effects	*Var.*		mR^2	0.19
Participants (intercept)	0.57	0.75	cR^2	0.62
False News Posts (intercept)	0.01	0.10		
Residual	0.5	0.71		

Supplementary Table 14. Results of linear mixed-effects models. The moderating role of cognitive reflection ($RQ_{\text{Cognitive reflection}}$) in the case of false news

**Accuracy**				
Fixed effects	*B*	*t*	*95% CI*	*p*
(Intercept)	2.42	35.93	2.29 – 2.55	<0.001
Treatment (low amplification)	-0.18	-12.01	-0.21 – -0.15	<0.001
Treatment (high amplification)	-0.02	-0.5	-0.09 – 0.05	0.618
Treatment (correction with link)	-0.12	-3.2	-0.19 – -0.05	0.001
CRT	-0.15	-4.01	-0.22 – -0.08	<0.001
Gender (male)	-0.09	-3.45	-0.15 – -0.04	0.001
Age (35-44)	0.01	0.18	-0.09 – 0.11	0.854
Age (45-54)	-0.11	-2.36	-0.20 – -0.02	0.018
Age (55-64)	-0.07	-1.4	-0.16 – 0.03	0.160
Age (65-75)	-0.12	-2.29	-0.21 – -0.02	0.022
Age (76+)	-0.33	-1.62	-0.73 – 0.07	0.104
Education (technical or vocational degree)	-0.07	-2.06	-0.13 – -0.00	0.040
Education (university degree)	-0.01	-0.24	-0.08 – 0.07	0.809
Treatment (low amplification):CRT	-0.01	-0.44	-0.04 – 0.02	0.66
Treatment (high amplification):CRT	0	0.04	-0.03 – 0.03	0.972
Treatment (correction with link):CRT	0.02	1.04	-0.01 – 0.05	0.299
Random effects	*Var.*		mR^2	0.07

Participants (intercept)	0.34	0.58	*cR*^{2}	0.52
False News Posts (intercept)	0.01	0.10		
Residual	0.37	0.61		
**Liking**				
Fixed effects	*B*	*t*	*95% CI*	*p*
(Intercept)	2.74	34.08	2.59 – 2.90	<0.001
Treatment (low amplification)	-0.08	-1.68	-0.17 – 0.01	0.092
Treatment (high amplification)	-0.18	-4.04	-0.27 – -0.10	<0.001
Treatment (correction with link)	-0.2	-4.4	-0.29 – -0.11	<0.001
CRT	-0.28	-14.19	-0.32 – -0.24	<0.001
Gender (male)	-0.19	-5.26	-0.27 – -0.12	<0.001
Age (35-44)	0	-0.06	-0.14 – 0.13	0.949
Age (45-54)	-0.1	-1.61	-0.23 – 0.02	0.108
Age (55-64)	-0.1	-1.51	-0.23 – 0.03	0.131
Age (65-75)	-0.21	-3.11	-0.35 – -0.08	0.002
Age (76+)	-0.41	-1.48	-0.96 – 0.13	0.139
Education (technical or vocational degree)	-0.06	-1.2	-0.14 – 0.03	0.229
Education (university degree)	-0.01	-0.14	-0.11 – 0.10	0.891
Treatment (low amplification):CRT	0.02	0.95	-0.02 – 0.06	0.341
Treatment (high amplification):CRT	0.03	1.46	-0.01 – 0.07	0.145
Treatment (correction with link):CRT	0.03	1.58	-0.01 – 0.07	0.113
Random effects	*Var.*		*mR*^{2}	0.08
Participants (intercept)	0.67	0.82	*cR*^{2}	0.59
False News Posts (intercept)	0.01	0.10		
Residual	0.54	0.73		
**Sharing**				
Fixed effects	*B*	*t*	*95% CI*	*p*
(Intercept)	2.72	32.91	2.55 – 2.88	<0.001
Treatment (low amplification)	-0.08	-1.9	-0.17 – 0.00	0.058
Treatment (high amplification)	-0.17	-3.79	-0.25 – -0.08	<0.001
Treatment (correction with link)	-0.19	-4.34	-0.28 – -0.10	<0.001
CRT	-0.29	-14.76	-0.33 – -0.26	<0.001
Gender (male)	-0.21	-5.63	-0.29 – -0.14	<0.001
Age (35-44)	0	-0.04	-0.14 – 0.14	0.969
Age (45-54)	-0.11	-1.6	-0.24 – 0.02	0.109
Age (55-64)	-0.09	-1.41	-0.23 – 0.04	0.158
Age (65-75)	-0.19	-2.75	-0.33 – -0.06	0.006
Age (76+)	-0.37	-1.29	-0.92 – 0.19	0.197
Education (technical or vocational degree)	0	0.07	-0.09 – 0.10	0.945
Education (university degree)	0.02	0.41	-0.08 – 0.13	0.683
Treatment (low amplification):CRT	0.02	1.23	-0.01 – 0.06	0.217
Treatment (high amplification):CRT	0.02	1.07	-0.02 – 0.06	0.283
Treatment (correction with link):CRT	0.04	1.94	-0.00 – 0.07	0.053
Random effects	*Var.*		*mR*^{2}	0.08

Participants (intercept)	0.72	0.85		cR^2	0.62
False News Posts (intercept)	0.01	0.10			
Residual	0.51	0.71			

Supplementary Table 15. Results of linear mixed-effects models. The moderating role of anti-expert sentiments ($RQ_{Anti\text{-}expert}$) in the case of true news

**Accuracy**				
Fixed effects	*B*	*t*	*95% CI*	*p*
(Intercept)	2.7	34.01	2.54 – 2.85	<0.001
Treatment (low amplification)	-0.10	-1.18	-0.27 – 0.07	0.239
Treatment (high amplification)	-0.15	-1.68	-0.32 – 0.02	0.092
Treatment (miscorrection with link)	-0.09	-1.04	-0.26 – 0.08	0.300
Anti-expert sentiments	-0.01	-0.47	-0.05 – 0.03	0.641
Gender (male)	-0.03	-1.37	-0.08 – 0.01	0.169
Age (35-44)	-0.18	-3.94	-0.27 – -0.09	<0.001
Age (45-54)	-0.28	-6.44	-0.36 – -0.19	<0.001
Age (55-64)	-0.27	-6.09	-0.35 – -0.18	<0.001
Age (65-75)	-0.29	-6.28	-0.38 – -0.20	<0.001
Age (76+)	-0.11	-0.61	-0.47 – 0.25	0.544
Education (technical or vocational degree)	0.08	2.70	0.02 – 0.14	0.007
Education (university degree)	0.11	3.22	0.04 – 0.18	0.001
Treatment (low amplification):Anti-expert sentiments	0.01	0.27	-0.05 – 0.06	0.787
Treatment (high amplification):Anti-expert sentiments	0.01	0.30	-0.05 – 0.06	0.761
Treatment (miscorrection with link):Anti-expert sentiments	0.01	0.24	-0.05 – 0.06	0.808
Random effects	*Var.*		mR^2	0.02
Participants (intercept)	0.21	0.46	cR^2	0.30
Residual	0.53	0.73		
**Liking**				
Fixed effects	*B*	*t*	*95% CI*	*p*
(Intercept)	2.23	19.45	2.01 – 2.46	<0.001
Treatment (low amplification)	-0.05	-0.41	-0.26 – 0.17	0.683
Treatment (high amplification)	-0.11	-1.00	-0.34 – 0.11	0.318
Treatment (miscorrection with link)	-0.06	-0.52	-0.28 – 0.16	0.604
Anti-expert sentiments	0.17	5.36	0.11 – 0.23	<0.001
Gender (male)	-0.05	-1.46	-0.13 – 0.02	0.145
Age (35-44)	-0.26	-3.59	-0.40 – -0.12	<0.001
Age (45-54)	-0.37	-5.55	-0.51 – -0.24	<0.001
Age (55-64)	-0.39	-5.65	-0.52 – -0.25	<0.001
Age (65-75)	-0.42	-5.82	-0.56 – -0.28	<0.001
Age (76+)	0.07	0.23	-0.49 – 0.62	0.819
Education (technical or vocational degree)	0.12	2.58	0.03 – 0.21	0.010
Education (university degree)	0.09	1.59	-0.02 – 0.19	0.113

Treatment (low amplification):Anti-expert sentiments	-0.01	-0.42	-0.08 – 0.05	0.678
Treatment (high amplification):Anti-expert sentiments	0.00	-0.09	-0.07 – 0.07	0.932
Treatment (miscorrection with link):Anti-expert sentiments	-0.02	-0.42	-0.09 – 0.06	0.673
Random effects	*Var.*		mR^2	0.02
Participants (intercept)	0.64	0.80	cR^2	0.44
Residual	0.85	0.92		
**Sharing**				
Fixed effects	*B*	*t*	*95% CI*	*p*
(Intercept)	1.96	17.15	1.73 – 2.18	<0.001
Treatment (low amplification)	-0.06	-0.56	-0.26 – 0.14	0.572
Treatment (high amplification)	-0.24	-2.26	-0.45 – -0.03	0.024
Treatment (miscorrection with link)	-0.11	-1.02	-0.32 – 0.10	0.310
Anti-expert sentiments	0.20	6.35	0.14 – 0.26	<0.001
Gender (male)	-0.08	-2.03	-0.15 – -0.00	0.043
Age (35-44)	-0.21	-2.88	-0.36 – -0.07	0.004
Age (45-54)	-0.28	-4.10	-0.42 – -0.15	<0.001
Age (55-64)	-0.28	-4.04	-0.42 – -0.15	<0.001
Age (65-75)	-0.32	-4.31	-0.46 – -0.17	<0.001
Age (76+)	0.04	0.12	-0.54 – 0.61	0.905
Education (technical or vocational degree)	0.14	2.93	0.05 – 0.24	0.003
Education (university degree)	0.03	0.53	-0.08 – 0.14	0.594
Treatment (low amplification):Anti-expert sentiments	-0.02	-0.53	-0.08 – 0.05	0.598
Treatment (high amplification):Anti-expert sentiments	0.04	1.05	-0.03 – 0.10	0.292
Treatment (miscorrection with link):Anti-expert sentiments	0.01	0.32	-0.06 – 0.08	0.748
Random effects	*Var.*		mR^2	0.03
Participants (intercept)	0.72	0.85	cR^2	0.51
Residual	0.73	0.85		

Supplementary Table 16. Results of linear mixed-effects models. The moderating role of susceptibility to social influence ($RQ_{\text{Social influence}}$) in the case of true news

**Accuracy**				
Fixed effects	*B*	*t*	*95% CI*	*p*
(Intercept)	2.26	36.63	2.14 – 2.38	<0.001
Treatment (low amplification)	-0.04	-0.74	-0.16 – 0.07	0.462
Treatment (high amplification)	-0.07	-1.23	-0.19 – 0.04	0.219
Treatment (miscorrection with link)	0.00	-0.03	-0.12 – 0.11	0.979
SSI	0.15	8.27	0.11 – 0.18	<0.001
Gender (male)	-0.02	-1.01	-0.07 – 0.02	0.313
Age (35-44)	-0.16	-3.62	-0.25 – -0.07	<0.001

Age (45-54)	-0.24	-5.70	-0.32 – -0.16	<0.001
Age (55-64)	-0.22	-5.23	-0.31 – -0.14	<0.001
Age (65-75)	-0.22	-4.84	-0.31 – -0.13	<0.001
Age (76+)	-0.06	-0.34	-0.41 – 0.29	0.731
Education (technical or vocational degree)	0.08	2.81	0.02 – 0.14	0.005
Education (university degree)	0.11	3.41	0.05 – 0.18	0.001
Treatment (low amplification):SSI	-0.01	-0.63	-0.06 – 0.03	0.528
Treatment (high amplification):SSI	-0.02	-0.91	-0.06 – 0.02	0.365
Treatment (miscorrection with link):SSI	-0.03	-1.24	-0.07 – 0.02	0.216
Random effects	*Var.*		*mR²*	0.04
Participants (intercept)	0.20	0.45	*cR²*	0.30
Residual	0.53	0.73		
**Liking**				
Fixed effects	*B*	*t*	*95% CI*	*p*
(Intercept)	1.64	18.62	1.47 – 1.81	<0.001
Treatment (low amplification)	0.02	0.31	-0.13 – 0.17	0.754
Treatment (high amplification)	-0.05	-0.66	-0.20 – 0.10	0.512
Treatment (miscorrection with link)	-0.05	-0.61	-0.19 – 0.10	0.544
SSI	0.40	16.31	0.36 – 0.45	<0.001
Gender (male)	-0.04	-1.13	-0.11 – 0.03	0.260
Age (35-44)	-0.17	-2.61	-0.30 – -0.04	0.009
Age (45-54)	-0.23	-3.73	-0.35 – -0.11	<0.001
Age (55-64)	-0.22	-3.46	-0.34 – -0.10	0.001
Age (65-75)	-0.18	-2.74	-0.31 – -0.05	0.006
Age (76+)	0.24	0.91	-0.28 – 0.76	0.365
Education (technical or vocational degree)	0.11	2.47	0.02 – 0.19	0.013
Education (university degree)	0.09	1.72	-0.01 – 0.18	0.085
Treatment (low amplification):SSI	-0.05	-1.63	-0.10 – 0.01	0.103
Treatment (high amplification):SSI	-0.03	-1.02	-0.09 – 0.03	0.308
Treatment (miscorrection with link):SSI	-0.02	-0.84	-0.08 – 0.03	0.400
Random effects	*Var.*		*mR²*	0.10
Participants (intercept)	0.52	0.72	*cR²*	0.44
Residual	0.85	0.92		
**Sharing**				
Fixed effects	*B*	*t*	*95% CI*	*p*
(Intercept)	1.35	15.53	1.18 – 1.53	<0.001
Treatment (low amplification)	-0.06	-0.79	-0.20 – 0.08	0.431
Treatment (high amplification)	-0.14	-2.04	-0.28 – -0.01	0.041
Treatment (miscorrection with link)	-0.10	-1.41	-0.24 – 0.04	0.158
SSI	0.43	18.00	0.39 – 0.48	<0.001
Gender (male)	-0.06	-1.74	-0.13 – 0.01	0.083
Age (35-44)	-0.11	-1.65	-0.24 – 0.02	0.099
Age (45-54)	-0.12	-1.9	-0.24 – 0.00	0.057
Age (55-64)	-0.09	-1.38	-0.21 – 0.04	0.168
Age (65-75)	-0.05	-0.71	-0.18 – 0.08	0.479
Age (76+)	0.23	0.87	-0.29 – 0.76	0.385

Education (technical or vocational degree)	0.12	2.78	0.04 – 0.21	0.006
Education (university degree)	0.03	0.55	-0.07 – 0.13	0.581
Treatment (low amplification):SSI	-0.02	-0.87	-0.08 – 0.03	0.386
Treatment (high amplification):SSI	0.01	0.26	-0.05 – 0.06	0.797
Treatment (miscorrection with link):SSI	0.01	0.39	-0.04 – 0.06	0.693
Random effects	*Var.*		*mR^2*	0.13
Participants (intercept)	0.57	0.75	*cR^2*	0.51
Residual	0.73	0.87		

Supplementary Table 17. Results of linear mixed-effects models. The moderating role of cognitive reflection ($RQ_{\text{Cognitive reflection}}$) in the case of true news

**Accuracy**				
Fixed effects	*B*	*t*	*95% CI*	*p*
(Intercept)	2.70	50.77	2.59 – 2.80	<0.001
Treatment (low amplification)	-0.04	-0.95	-0.13 – 0.04	0.342
Treatment (high amplification)	-0.07	-1.45	-0.15 – 0.02	0.146
Treatment (miscorrection with link)	-0.09	-2.11	-0.18 – -0.01	0.035
CRT	-0.02	-0.95	-0.05 – 0.02	0.343
Gender (male)	-0.04	-1.81	-0.09 – 0.00	0.071
Age (35-44)	-0.18	-3.97	-0.27 – -0.09	<0.001
Age (45-54)	-0.28	-6.46	-0.36 – -0.19	<0.001
Age (55-64)	-0.27	-6.18	-0.35 – -0.18	<0.001
Age (65-75)	-0.29	-6.31	-0.38 – -0.20	<0.001
Age (76+)	-0.12	-0.64	-0.47 – 0.24	0.524
Education (technical or vocational degree)	0.09	2.89	0.03 – 0.15	0.004
Education (university degree)	0.12	3.45	0.05 – 0.19	0.001
Treatment (low amplification):CRT	-0.02	-0.93	-0.06 – 0.02	0.355
Treatment (high amplification):CRT	-0.03	-1.45	-0.07 – 0.01	0.147
Treatment (miscorrection with link):CRT	0.01	0.65	-0.03 – 0.05	0.517
Random effects	*Var.*		*mR^2*	0.02
Participants (intercept)	0.21	0.46	*cR^2*	0.30
Residual	0.53	0.73		
**Liking**				
Fixed effects	*B*	*t*	*95% CI*	*p*
(Intercept)	3.04	38.51	2.89 – 3.20	<0.001
Treatment (low amplification)	0.00	-0.06	-0.12 – 0.11	0.954
Treatment (high amplification)	0.00	0.01	-0.11 – 0.11	0.990
Treatment (miscorrection with link)	-0.11	-1.94	-0.23 – 0.00	0.053
CRT	-0.14	-6.41	-0.19 – -0.10	<0.001
Gender (male)	-0.14	-3.69	-0.21 – -0.07	<0.001
Age (35-44)	-0.23	-3.27	-0.37 – -0.09	0.001
Age (45-54)	-0.35	-5.25	-0.48 – -0.22	<0.001
Age (55-64)	-0.36	-5.27	-0.49 – -0.22	<0.001
Age (65-75)	-0.38	-5.36	-0.52 – -0.24	<0.001
Age (76+)	0.06	0.22	-0.49 – 0.61	0.826

Education (technical or vocational degree)	0.15	3.26	0.06 – 0.24	0.001
Education (university degree)	0.13	2.50	0.03 – 0.24	0.013
Treatment (low amplification):CRT	-0.04	-1.74	-0.09 – 0.01	0.082
Treatment (high amplification):CRT	-0.06	-2.44	-0.11 – -0.01	0.015
Treatment (miscorrection with link):CRT	0.00	0.18	-0.04 – 0.05	0.854
Random effects	*Var.*		mR^2	0.04
Participants (intercept)	0.62	0.79	cR^2	0.45
Residual	0.85	0.92		
**Sharing**				
Fixed effects	*B*	*t*	*95% CI*	*p*
(Intercept)	2.88	36.02	2.72 – 3.04	<0.001
Treatment (low amplification)	-0.05	-0.98	-0.16 – 0.05	0.328
Treatment (high amplification)	-0.01	-0.24	-0.12 – 0.09	0.812
Treatment (miscorrection with link)	-0.03	-0.53	-0.13 – 0.08	0.597
CRT	-0.16	-7.20	-0.20 – -0.12	<0.001
Gender (male)	-0.17	-4.40	-0.25 – -0.09	<0.001
Age (35-44)	-0.18	-2.43	-0.32 – -0.03	0.015
Age (45-54)	-0.25	-3.70	-0.39 – -0.12	<0.001
Age (55-64)	-0.24	-3.51	-0.38 – -0.11	<0.001
Age (65-75)	-0.27	-3.71	-0.41 – -0.13	<0.001
Age (76+)	0.04	0.12	-0.53 – 0.61	0.903
Education (technical or vocational degree)	0.17	3.57	0.08 – 0.27	<0.001
Education (university degree)	0.08	1.45	-0.03 – 0.19	0.147
Treatment (low amplification):CRT	-0.03	-1.27	-0.08 – 0.02	0.202
Treatment (high amplification):CRT	-0.06	-2.48	-0.10 – -0.01	0.013
Treatment (miscorrection with link):CRT	-0.02	-0.94	-0.07 – 0.02	0.349
Random effects	*Var.*		mR^2	0.04
Participants (intercept)	0.71	0.84	cR^2	0.51
Residual	0.73	0.85		

Note. The mixed-effects regressions for $RQ_{\text{Anti-expert}}$ were run on 10,183 observations (perceived accuracy), 10,191 observations (like), and 10,184 observations (share) with 2,455 respondents and nine false news posts. The mixed-effects regressions for $RQ_{\text{Social influence}}$ were run on 10,198 observations (perceived accuracy), 10,206 observations (like), and 10,199 observations (share) with 2,458 respondents and nine false news posts. The mixed-effects regressions for $RQ_{\text{Cognitive reflection}}$ were run on 10,201 observations (perceived accuracy), 10,209 observations (like), and 10,202 observations (share) with 2,459 respondents and nine false news posts.

The mixed-effects regressions for $RQ_{\text{Anti-expert}}$ were run on 9,759 observations (perceived accuracy), 9,757 observations (like), and 9,759 observations (share) with 2,455 respondents and four true news posts. The mixed-effects regressions for $RQ_{\text{Social influence}}$ were run on 9,771 observations (perceived accuracy), 9,769 observations (like), and 9,771 observations (share) with 2,444 respondents and four false news posts. The mixed-effects regressions for

$RQ_{Cognitive\ reflection}$ were run on 9,773 observations (perceived accuracy), 9,773 observations (like), and 9,775 observations (share) with 2,445 respondents and four true news posts. As we showed the same four true news posts to all our respondents, we simplified the structure of our mixed-effects regressions by removing the random intercept for social media posts. That is, we computed the following model: response ~ social miscorrection treatment:individual differences + gender + age + education + (1|respondent id). More detailed analyses (including our code and data) can be found on OSF (files analysis_fakenews_osf and analysis_truenews_osf).

Supplementary Table 18. Results of linear mixed-effects models for $H_{Correct\ false}$ in Italy excluding respondents that failed attention checks.

**Perceived accuracy**				
Fixed effects	*B*	*t*	*95% CI*	*p*
(Intercept)	2.00	32.04	1.88 – 2.12	<0.001
Treatment (low amplification)	-0.04	-2.00	-0.08 – -0.00	0.045
Treatment (high amplification)	-0.13	-6.65	-0.17 – -0.09	<0.001
Treatment (correction with link)	-0.12	-6.33	-0.16 – -0.08	<0.001
Gender (male)	0.01	0.29	-0.05 – 0.06	0.775
Age (35-44)	0.02	0.38	-0.08 – 0.13	0.701
Age (45-54)	-0.09	-1.83	-0.19 – 0.01	0.067
Age (55-64)	-0.05	-0.89	-0.15 – 0.05	0.371
Age (65-75)	-0.09	-1.71	-0.20 – 0.01	0.088
Age (76+)	-0.26	-1.24	-0.67 – 0.15	0.214
Education (technical or vocational degree)	-0.13	-3.58	-0.19 – -0.06	<0.001
Education (university degree)	-0.09	-2.20	-0.17 – -0.01	0.028
Random effects	*Variance*	*SD*	mR^2	0.01
Participants (intercept)	0.37	0.61	cR^2	0.52
False News Posts (intercept)	0.01	0.10		
Residual	0.36	0.60		
**Liking**				
Fixed effects	*B*	*t*	*95% CI*	*p*
(Intercept)	2.07	28.21	1.93 – 2.22	<0.001
Treatment (low amplification)	-0.04	-1.78	-0.09 – 0.00	0.075
Treatment (high amplification)	-0.12	-5.32	-0.17 – -0.08	<0.001
Treatment (correction with link)	-0.14	-5.87	-0.18 – -0.09	<0.001
Gender (male)	-0.05	-1.39	-0.13 – 0.02	0.164
Age (35-44)	0.03	0.42	-0.11 – 0.17	0.674
Age (45-54)	-0.06	-0.82	-0.19 – 0.08	0.410
Age (55-64)	-0.06	-0.79	-0.19 – 0.08	0.431
Age (65-75)	-0.17	-2.25	-0.31 – -0.02	0.024
Age (76+)	-0.30	-1.03	-0.86 – 0.27	0.305
Education (technical or vocational degree)	-0.14	-2.86	-0.23 – -0.04	0.004
Education (university degree)	-0.12	-2.20	-0.23 – -0.01	0.028
Random effects	*Variance*	*SD*	mR^2	0.01
Participants (intercept)	0.73	0.85	cR^2	0.58
False News Posts (intercept)	0.01	0.10		
Residual	0.53	0.73		
**Sharing**				

Fixed effects	*B*	*t*	*95% CI*	*p*
(Intercept)	2.01	26.72	1.86 – 2.16	<0.001
Treatment (low amplification)	-0.04	-1.94	-0.09 – 0.00	0.052
Treatment (high amplification)	-0.13	-5.77	-0.18 – -0.09	<0.001
Treatment (correction with link)	-0.12	-5.28	-0.16 – -0.08	<0.001
Gender (male)	-0.06	-1.47	-0.13 – 0.02	0.142
Age (35-44)	0.04	0.50	-0.11 – 0.18	0.618
Age (45-54)	-0.05	-0.73	-0.19 – 0.09	0.465
Age (55-64)	-0.05	-0.73	-0.19 – 0.09	0.466
Age (65-75)	-0.14	-1.84	-0.29 – 0.01	0.065
Age (76+)	-0.24	-0.82	-0.82 – 0.33	0.412
Education (technical or vocational degree)	-0.08	-1.60	-0.17 – 0.02	0.109
Education (university degree)	-0.11	-1.91	-0.22 – 0.00	0.056
Random effects	*Variance*	*SD*	mR^2	0.01
Participants (intercept)	0.77	0.89	cR^2	0.61
False News Posts (intercept)	0.01	0.10		
Residual	0.50	0.71		

Note. The mixed-effects regressions were run on 9,763 observations (perceived accuracy), 9,771 observations (like), and 9,764 observations (share) with 2,351respondents and 9 false news posts. mR^2 = marginal R^2 (i.e., variance of the fixed effects); cR^2 = conditional R^2 (i.e., variance of the fixed and random effects). Reference groups: Treatment = control condition; gender = female; age = 18-24; education = less than primary education.

Supplementary Table 19. Results of linear mixed-effects models for $H_{Correct\ false}$ in Italy controlling for congeniality.

**Perceived accuracy**				
Fixed effects	*B*	*t*	*95% CI*	*p*
(Intercept)	2.00	33.63	1.89 – 2.12	<0.001
Treatment (low amplification)	-0.03	-1.72	-0.07 – 0.00	0.085
Treatment (high amplification)	-0.12	-6.25	-0.15 – -0.08	<0.001
Treatment (correction with link)	-0.12	-6.16	-0.15 – -0.08	<0.001
Gender (male)	-0.02	-0.55	-0.07 – 0.04	0.585
Age (35-44)	0.01	0.26	-0.09 – 0.12	0.794
Age (45-54)	-0.10	-2.05	-0.20 – -0.00	0.040
Age (55-64)	-0.06	-1.22	-0.16 – 0.04	0.223
Age (65-75)	-0.12	-2.38	-0.23 – -0.02	0.017
Age (76+)	-0.30	-1.42	-0.72 – 0.11	0.155
Education (technical or vocational degree)	-0.12	-3.49	-0.19 – -0.05	<0.001
Education (university degree)	-0.07	-1.84	-0.15 – 0.00	0.066
Congeniality (congenial)	0.09	5.14		
Random effects	*Variance*	*SD*	mR^2	0.01
Participants (intercept)	0.37	0.61	cR^2	0.52
False News Posts (intercept)	0.01	0.10		
Residual	0.37	0.651		
**Liking**				
Fixed effects	*B*	*t*	*95% CI*	*p*
(Intercept)	2.11	29.24	1.97 – 2.25	<0.001
Treatment (low amplification)	-0.04	-1.69	-0.08 – 0.01	0.091

Treatment (high amplification)	-0.13	-5.50	-0.17 – -0.08	<0.001
Treatment (correction with link)	-0.14	-6.01	-0.18 – -0.09	<0.001
Gender (male)	-0.08	-2.11	-0.15 – -0.01	0.035
Age (35-44)	0.00	0.04	-0.14 – 0.14	0.970
Age (45-54)	-0.09	-1.29	-0.22 – 0.05	0.198
Age (55-64)	-0.09	-1.29	-0.22 – 0.05	0.198
Age (65-75)	-0.23	-3.15	-0.37 – -0.09	0.002
Age (76+)	-0.37	-1.26	-0.94 – 0.20	0.207
Education (technical or vocational degree)	-0.13	-2.77	-0.23 – -0.04	0.006
Education (university degree)	-0.10	-1.83	-0.21 – 0.01	0.068
Congeniality (congenial)	0.11	5.12	0.07 – 0.15	<0.001
Random effects	*Variance*	*SD*	mR^2	0.01
Participants (intercept)	0.75	0.87	cR^2	0.59
False News Posts (intercept)	0.01	0.10		
Residual	0.54	0.73		
**Sharing**				
Fixed effects	*B*	*t*	*95% CI*	*p*
(Intercept)	2.06	27.64	1.91 – 2.21	<0.001
Treatment (low amplification)	-0.04	-1.64	-0.08 – 0.01	0.101
Treatment (high amplification)	-0.13	-5.69	-0.17 – -0.08	<0.001
Treatment (correction with link)	-0.12	-5.28	-0.16 – -0.07	<0.001
Gender (male)	-0.09	-2.41	-0.17 – -0.02	0.016
Age (35-44)	0.01	0.07	-0.14 – 0.15	0.944
Age (45-54)	-0.09	-1.27	-0.22 – 0.05	0.205
Age (55-64)	-0.08	-1.18	-0.22 – 0.05	0.237
Age (65-75)	-0.21	-2.80	-0.35 – -0.06	0.005
Age (76+)	-0.32	-1.08	-0.91 – 0.26	0.282
Education (technical or vocational degree)	-0.08	-1.60	-0.17 – 0.02	0.109
Education (university degree)	-0.08	-1.35	-0.19 – 0.03	0.178
Congeniality (congenial)	0.09	4.62	0.05 – 0.13	<0.001
Random effects	*Variance*	*SD*	mR^2	0.01
Participants (intercept)	0.81	0.90	cR^2	0.62
False News Posts (intercept)	0.01	0.10		
Residual	0.51	0.71		

Note. The mixed-effects regressions were run on 10,198 observations (perceived accuracy), 10,206 observations (like), and 10,199 observations (share) with 2,459 respondents and 9 false news posts. mR^2 = marginal R^2 (i.e., variance of the fixed effects); cR^2 = conditional R^2 (i.e., variance of the fixed and random effects). Reference groups: Treatment = control condition; gender = female; age = 18-24; education = less than primary education.

Supplementary Table 20. Results of linear mixed-effects models for $H_{\text{Miscorrect true}}$ in Italy excluding respondents that failed attention checks.

**Perceived accuracy**				
Fixed effects	*B*	*t*	*95% CI*	*p*
(Intercept)	2.67	62.02	2.59 – 2.76	<0.001
Treatment (low amplification)	-0.08	-3.39	-0.12 – -0.03	0.001
Treatment (high amplification)	-0.13	-5.67	-0.18 – -0.09	<0.001
Treatment (miscorrection with link)	-0.07	-3.25	-0.12 – -0.03	0.001

Gender (male)	-0.02	-0.92	-0.07 – 0.03	0.358
Age (35-44)	-0.20	-4.36	-0.30 – -0.11	<0.001
Age (45-54)	-0.29	-6.57	-0.38 – -0.20	<0.001
Age (55-64)	-0.29	-6.49	-0.38 – -0.20	<0.001
Age (65-75)	-0.30	-6.33	-0.39 – -0.20	<0.001
Age (76+)	-0.12	-0.65	-0.47 – 0.24	0.519
Education (technical or vocational degree)	0.08	2.64	0.02 – 0.14	0.008
Education (university degree)	0.11	3.02	0.04 – 0.17	0.003
Random effects	*Variance*	*SD*	mR^2	0.02
Participants (intercept)	0.22	0.47	cR^2	0.30
Residual	0.53	0.73		
**Liking**				
Fixed effects	*B*	*t*	*95% CI*	*p*
(Intercept)	2.72	40.75	2.59 – 2.85	<0.001
Treatment (low amplification)	-0.09	-3.05	-0.15 – -0.03	0.002
Treatment (high amplification)	-0.13	-4.42	-0.19 – -0.07	<0.001
Treatment (miscorrection with link)	-0.11	-3.57	-0.16 – -0.05	<0.001
Gender (male)	-0.04	-1.06	-0.12 – 0.03	0.291
Age (35-44)	-0.24	-3.32	-0.39 – -0.10	0.001
Age (45-54)	-0.34	-4.97	-0.48 – -0.21	<0.001
Age (55-64)	-0.36	-5.15	-0.50 – -0.22	<0.001
Age (65-75)	-0.38	-5.20	-0.53 – -0.24	<0.001
Age (76+)	0.11	0.38	-0.45 – 0.67	0.707
Education (technical or vocational degree)	0.10	2.18	0.01 – 0.20	0.029
Education (university degree)	0.05	0.90	-0.06 – 0.16	0.366
Random effects	*Variance*	*SD*	mR^2	0.01
Participants (intercept)	0.65	0.81	cR^2	0.44
Residual	0.85	0.92		
**Sharing**				
Fixed effects	*B*	*t*	*95% CI*	*p*
(Intercept)	2.52	36.75	2.38 – 2.65	<0.001
Treatment (low amplification)	-0.11	-4.16	-0.17 – -0.06	<0.001
Treatment (high amplification)	-0.14	-4.95	-0.19 – -0.08	<0.001
Treatment (miscorrection with link)	-0.08	-2.79	-0.13 – -0.02	0.005
Gender (male)	-0.07	-1.68	-0.14 – 0.01	0.093
Age (35-44)	-0.19	-2.48	-0.34 – -0.04	0.013
Age (45-54)	-0.25	-3.44	-0.39 – -0.11	0.001
Age (55-64)	-0.24	-3.36	-0.39 – -0.10	0.001
Age (65-75)	-0.27	-3.58	-0.42 – -0.12	<0.001
Age (76+)	0.09	0.30	-0.49 – 0.67	0.768
Education (technical or vocational degree)	0.12	2.43	0.02 – 0.22	0.015
Education (university degree)	-0.01	-0.14	-0.12 – 0.10	0.892
Random effects	*Variance*	*SD*	mR^2	0.01
Participants (intercept)	0.74	0.86	cR^2	0.51
Residual	0.72	0.85		

Note. The mixed-effects regressions were run on 9,400 observations (perceived accuracy), 9,398 observations (like), and 9,399 observations (share) with 2,351 respondents and 4 true news posts. mR^2 = marginal R^2 (i.e., variance of the fixed effects); cR^2 = conditional R^2 (i.e., variance of the fixed and random effects). Reference groups: Treatment = control condition; gender = female; age = 18-24; education = less than primary education.

Supplementary Table 21. Results of linear mixed-effects models for $H_{\text{Miscorrect true}}$ in Italy controlling for congeniality.

**Perceived accuracy**				
Fixed effects	*B*	*t*	*95% CI*	*p*
(Intercept)	2.48	54.44	2.39 – 2.57	<0.001
Treatment (low amplification)	-0.08	-3.55	-0.12 – -0.04	<0.001
Treatment (high amplification)	-0.12	-5.51	-0.17 – -0.08	<0.001
Treatment (miscorrection with link)	-0.07	-3.10	-0.11 – -0.03	0.002
Gender (male)	-0.03	-1.44	-0.08 – 0.01	0.149
Age (35-44)	-0.17	-3.85	-0.26 – -0.08	<0.001
Age (45-54)	-0.27	-6.36	-0.35 – -0.19	<0.001
Age (55-64)	-0.27	-6.20	-0.35 – -0.18	<0.001
Age (65-75)	-0.29	-6.44	-0.38 – -0.20	<0.001
Age (76+)	-0.13	-0.70	-0.47 – 0.22	0.482
Education (technical or vocational degree)	0.08	2.61	0.02 – 0.13	0.009
Education (university degree)	0.11	3.17	0.04 – 0.17	0.002
Congeniality (congenial)	0.22	9.87	0.18 – 0.27	<0.001
Random effects	*Variance*	*SD*	mR^2	0.03
Participants (intercept)	0.20	0.47	cR^2	0.30
Residual	0.53	0.73		
**Liking**				
Fixed effects	*B*	*t*	*95% CI*	*p*
(Intercept)	2.54	36.46	2.40 – 2.67	<0.001
Treatment (low amplification)	-0.09	-3.21	-0.15 – -0.04	0.001
Treatment (high amplification)	-0.13	-4.33	-0.18 – -0.07	<0.001
Treatment (miscorrection with link)	-0.11	-3.61	-0.16 – -0.05	<0.001
Gender (male)	-0.06	-1.72	-0.14 – 0.01	0.085
Age (25-34)	-0.21	-3.01	-0.35 – -0.07	0.003
Age (35-44)	-0.33	-4.90	-0.46 – -0.20	<0.001
Age (45-54)	-0.34	-5.03	-0.48 – -0.21	<0.001
Age (55-64)	-0.39	-5.37	-0.53 – -0.24	<0.001
Age (65+)	0.08	0.27	-0.48 – 0.64	0.788
Education (technical or vocational degree)	0.10	2.09	0.01 – 0.19	0.036
Education (university degree)	0.07	1.31	-0.03 – 0.18	0.190
Congeniality (congenial)	0.24	7.98	0.18 – 0.30	<0.001
Random effects	*Variance*	*SD*	mR^2	0.02
Participants (intercept)	0.64	0.80	cR^2	0.44
Residual	0.85	0.92		
**Sharing**				
Fixed effects	*B*	*t*	*95% CI*	*p*
(Intercept)	2.35	33.25	2.22 – 2.49	<0.001
Treatment (low amplification)	-0.11	-4.21	-0.17 – -0.06	<0.001
Treatment (high amplification)	-0.13	-4.90	-0.19 – -0.08	<0.001
Treatment (miscorrection with link)	-0.07	-2.75	-0.13 – -0.02	0.006
Gender (male)	-0.09	-2.31	-0.17 – -0.01	0.021
Age (25-34)	-0.16	-2.16	-0.31 – -0.01	0.031
Age (35-44)	-0.23	-3.33	-0.37 – -0.10	0.001

Age (45-54)	-0.23	-3.26	-0.37 – -0.09	0.001
Age (55-64)	-0.28	-3.73	-0.42 – -0.13	<0.001
Age (65+)	0.05	0.18	-0.53 – 0.63	0.856
Education (technical or vocational degree)	0.11	2.31	0.02 – 0.21	0.021
Education (university degree)	0.01	0.20	-0.10 – 0.12	0.840
Congeniality (congenial)	0.22	7.84	0.17 – 0.28	<0.001
Random effects	*Variance*	*SD*	mR^2	0.01
Participants (intercept)	0.74	0.86	cR^2	0.51
Residual	0.73	0.85		

Note. The mixed-effects regressions were run on 9,400 observations (perceived accuracy), 9,769 observations (like), and 9,771 observations (share) with 2445 respondents and 4 true news posts. mR^2 = marginal R^2 (i.e., variance of the fixed effects); cR^2 = conditional R^2 (i.e., variance of the fixed and random effects). Reference groups: Treatment = control condition; gender = female; age = 18-24; education = less than primary education.

Supplementary Table 22. Overview of demographics of the Italy Experiment.

Demographic Variable	Frequency	Percentage (%)
Gender		
Male	1212	49.1
Female	1247	50.5
Non-binary / third gender / other	8	0.3
Age		
18-24	279	11.3
25-34	459	18.6
35-44	662	26.8
45-54	599	24.3
55-64	457	18.5
65+	11	0.4
Education		
Low	1552	62.9
Mid	375	15.2
High	540	21.9
Party		
Fdl	381	15.4
FI	196	7.9
Lega	210	8.5
M5S	385	15.6
PD	359	14.6
No PID	691	28.0
Other party	245	9.9

Note. Education: low = incomplete Secondary Education, Secondary Education Completed, or Secondary Education Completed; mid = some Vocational or Technical Qualifications or Vocational or Technical Qualifications Completed; high = University Education Completed (First Degree e.g., BA, BSc), Postgraduate Education Completed (e.g., Masters), Doctorate, or Post-doctorate or equivalent. Party: No PID = *no party* and *don't know* option. Note that "Other party" includes one NA.

Supplementary Methods

Germany Experiment (Experiment 3)

The following table gives an overview of the detailed results of the interaction of social corrections with anti-expert sentiments ($RQ_{Anti\text{-}expert}$), susceptibility to social influence ($RQ_{Social\ influence}$), and cognitive reflection ($RQ_{Cognitive\ reflection}$).

Supplementary Table 23. Results of linear mixed-effects models. The moderating role of anti-expert sentiments ($RQ_{Anti\text{-}expert}$) in the case of false news.

**Accuracy**				
Fixed effects	*B*	*t*	*95% CI*	*p*
(Intercept)	1.53	15.31	1.33 – 1.73	<0.001
Treatment (low amplification)	-0.12	-1.44	-0.29 – 0.04	0.149
Treatment (high amplification)	-0.02	-0.18	-0.18 – 0.15	0.854
Treatment (correction with link)	-0.07	-0.88	-0.24 – 0.09	0.378
Anti-expert sentiments	0.34	15.10	0.30 – 0.39	<0.001
Gender (male)	-0.04	-1.60	-0.10 – 0.01	0.109
Age (25-34)	-0.09	-1.56	-0.20 – 0.02	0.118
Age (35-44)	-0.12	-2.21	-0.22 – -0.01	0.027
Age (45-54)	-0.17	-3.20	-0.27 – -0.06	0.001
Age (55-64)	-0.25	-4.78	-0.35 – -0.15	<0.001
Age (65+)	-0.27	-4.85	-0.38 – -0.16	<0.001
Education (technical or vocational degree)	-0.17	-3.99	-0.25 – -0.08	<0.001
Education (university degree)	-0.13	-3.36	-0.21 – -0.06	0.001
Treatment (low amplification):Anti-expert sentiments	0.01	0.24	-0.05 – 0.06	0.814
Treatment (high amplification):Anti-expert sentiments	-0.04	-1.57	-0.10 – 0.01	0.117
Treatment (correction with link):Anti-expert sentiments	-0.03	-1.04	-0.08 – 0.03	0.298
Random effects	*Var.*		mR^2	0.13
Participants (intercept)	0.26	0.51	cR^2	0.48
False News Posts (intercept)	0.02	0.14		
Residual	0.43	0.66		
**Liking**				
Fixed effects	*B*	*t*	*95% CI*	*p*
(Intercept)	1.26	11.28	1.04 – 1.48	<0.001
Treatment (low amplification)	0.13	1.48	-0.04 – 0.31	0.140
Treatment (high amplification)	0.04	0.40	-0.14 – 0.21	0.689
Treatment (correction with link)	0.08	0.93	-0.09 – 0.26	0.355
Anti-expert sentiments	0.48	16.70	0.42 – 0.53	<0.001

Gender (male)	0.10	2.51	0.02 – 0.18	0.012
Age (25-34)	-0.11	-1.30	-0.27 – 0.05	0.193
Age (35-44)	-0.27	-3.64	-0.42 – -0.13	<0.001
Age (45-54)	-0.51	-6.76	-0.65 – -0.36	<0.001
Age (55-64)	-0.62	-8.25	-0.76 – -0.47	<0.001
Age (65+)	-0.55	-6.92	-0.71 – -0.40	<0.001
Education (technical or vocational degree)	-0.34	-5.56	-0.45 – -0.22	<0.001
Education (university degree)	-0.24	-4.16	-0.35 – -0.13	<0.001
Treatment (low amplification):Anti-expert sentiments	-0.08	-2.73	-0.14 – -0.02	0.006
Treatment (high amplification):Anti-expert sentiments	-0.05	-1.50	-0.10 – 0.01	0.133
Treatment (correction with link):Anti-expert sentiments	-0.08	-2.54	-0.14 – -0.02	0.011
Random effects	*Var.*		*mR²*	0.17
Participants (intercept)	0.69	0.83	*cR²*	0.67
False News Posts (intercept)	0.00	0.00		
Residual	0.46	0.68		
**Sharing**				
Fixed effects	*B*	*t*	*95% CI*	*p*
(Intercept)	1.32	11.76	1.10 – 1.54	<0.001
Treatment (low amplification)	0.05	0.6	-0.11 – 0.21	0.548
Treatment (high amplification)	0.07	0.83	-0.09 – 0.23	0.406
Treatment (correction with link)	0.10	1.18	-0.06 – 0.26	0.236
Anti-expert sentiments	0.48	16.85	0.42 – 0.53	<0.001
Gender (male)	0.14	3.26	0.05 – 0.22	0.001
Age (25-34)	-0.16	-1.85	-0.33 – 0.01	0.065
Age (35-44)	-0.33	-4.16	-0.48 – -0.17	<0.001
Age (45-54)	-0.62	-7.90	-0.77 – -0.46	<0.001
Age (55-64)	-0.74	-9.53	-0.90 – -0.59	<0.001
Age (65+)	-0.71	-8.58	-0.88 – -0.55	<0.001
Education (technical or vocational degree)	-0.35	-5.59	-0.48 – -0.23	<0.001
Education (university degree)	-0.26	-4.42	-0.38 – -0.15	<0.001
Treatment (low amplification):Anti-expert sentiments	-0.05	-1.68	-0.10 – 0.01	0.093
Treatment (high amplification):Anti-expert sentiments	-0.05	-1.92	-0.11 – 0.00	0.055
Treatment (amplification + link):Anti-expert sentiments	-0.08	-2.74	-0.13 – -0.02	0.006
Random effects	*Var.*		*mR²*	0.18
Participants (intercept)	0.80	0.89	*cR²*	0.74
False News Posts (intercept)	0.00	0.00		
Residual	0.38	0.62		

Supplementary Table 24. Results of linear mixed-effects models. The moderating role of susceptibility to social influence ($RQ_{Social\ influence}$) in the case of false news.

**Accuracy**				
Fixed effects	*B*	*t*	*95% CI*	*p*
(Intercept)	1.63	17.49	1.45 – 1.82	<0.001
Treatment (low amplification)	-0.12	-2.00	-0.24 – -0.00	0.046
Treatment (high amplification)	-0.17	-2.78	-0.29 – -0.05	0.005
Treatment (correction with link)	-0.17	-2.77	-0.29 – -0.05	0.006
SSI	0.29	14.20	0.25 – 0.33	<0.001
Gender (male)	-0.03	-1.06	-0.08 – 0.03	0.288
Age (25-34)	0.02	0.27	-0.10 – 0.13	0.787
Age (35-44)	0.02	0.29	-0.09 – 0.12	0.773
Age (45-54)	0.04	0.80	-0.06 – 0.15	0.426
Age (55-64)	0.01	0.28	-0.09 – 0.12	0.781
Age (65+)	0.02	0.38	-0.09 – 0.13	0.708
Education (technical or vocational degree)	-0.12	-2.94	-0.21 – -0.04	0.003
Education (university degree)	-0.08	-2.03	-0.16 – -0.00	0.042
Treatment (low amplification):SSI	0.01	0.29	-0.04 – 0.06	0.772
Treatment (high amplification):SSI	0.01	0.49	-0.04 – 0.06	0.624
Treatment (correction with link):SSI	0.00	0.15	-0.04 – 0.05	0.882
Random effects	*Var.*		mR^2	0.13
Participants (intercept)	0.27	0.52	cR^2	0.48
False News Posts (intercept)	0.02	0.14		
Residual	0.43	0.66		
**Liking**				
Fixed effects	*B*	*t*	*95% CI*	*p*
(Intercept)	1.05	10.57	0.86 – 1.24	<0.001
Treatment (low amplification)	0.02	0.31	-0.11 – 0.15	0.756
Treatment (high amplification)	-0.13	-2.07	-0.26 – -0.01	0.038
Treatment (correction with link)	-0.09	-1.42	-0.22 – 0.03	0.155
SSI	0.53	21.18	0.48 – 0.58	<0.001
Gender (male)	0.11	2.96	0.04 – 0.19	0.003
Age (25-34)	0.06	0.72	-0.10 – 0.21	0.473
Age (35-44)	-0.06	-0.85	-0.20 – 0.08	0.397
Age (45-54)	-0.15	-2.12	-0.29 – -0.01	0.034
Age (55-64)	-0.16	-2.18	-0.30 – -0.02	0.029
Age (65+)	-0.04	-0.54	-0.19 – 0.11	0.586
Education (technical or vocational degree)	-0.24	-4.22	-0.35 – -0.13	<0.001
Education (university degree)	-0.14	-2.67	-0.25 – -0.04	0.008
Treatment (low amplification):SSI	-0.06	-2.10	-0.11 – -0.00	0.036
Treatment (high amplification):SSI	0.02	0.70	-0.03 – 0.07	0.483
Treatment (correction with link):SSI	-0.02	-0.77	-0.07 – 0.03	0.443
Random effects	*Var.*		mR^2	0.24
Participants (intercept)	0.59	0.77	cR^2	0.67
False News Posts (intercept)	0.00	0.00		

Residual	0.46	0.68		
**Sharing**				
Fixed effects	*B*	*t*	*95% CI*	*p*
(Intercept)	1.02	10.2	0.82 – 1.22	<0.001
Treatment (low amplification)	-0.02	-0.41	-0.14 – 0.09	0.679
Treatment (high amplification)	-0.08	-1.40	-0.20 – 0.03	0.162
Treatment (correction with link)	-0.03	-0.51	-0.15 – 0.09	0.612
SSI	0.56	22.72	0.51 – 0.61	<0.001
Gender (male)	0.15	3.77	0.07 – 0.22	<0.001
Age (25-34)	0.01	0.13	-0.15 – 0.17	0.897
Age (35-44)	-0.10	-1.38	-0.25 – 0.04	0.168
Age (45-54)	-0.24	-3.30	-0.39 – -0.10	0.001
Age (55-64)	-0.26	-3.45	-0.41 – -0.11	0.001
Age (65+)	-0.17	-2.16	-0.33 – -0.02	0.031
Education (technical or vocational degree)	-0.25	-4.22	-0.36 – -0.13	<0.001
Education (university degree)	-0.16	-2.94	-0.27 – -0.05	0.003
Treatment (low amplification):SSI	-0.03	-1.12	-0.07 – 0.02	0.264
Treatment (high amplification):SSI	0.00	0.01	-0.05 – 0.05	0.990
Treatment (correction with link):SSI	-0.04	-1.69	-0.09 – 0.01	0.092
Random effects	*Var.*		mR^2	0.27
Participants (intercept)	0.67	0.82	cR^2	0.74
False News Posts (intercept)	0.00	0.00		
Residual	0.38	0.62		

Supplementary Table 25. Results of linear mixed-effects models. The moderating role of cognitive reflection ($RQ_{Cognitive\ reflection}$) in the case of false news.

**Accuracy**				
Fixed effects	*B*	*t*	*95% CI*	*p*
(Intercept)	2.65	32.16	2.49 – 2.82	<0.001
Treatment (low amplification)	-0.19	-4.46	-0.27 – -0.10	<0.001
Treatment (high amplification)	-0.14	-3.34	-0.22 – -0.06	0.001
Treatment (correction with link)	-0.18	-4.44	-0.27 – -0.10	<0.001
CRT	-0.12	-6.40	-0.15 – -0.08	<0.001
Gender (male)	0.03	0.90	-0.03 – 0.09	0.368
Age (25-34)	-0.06	-0.92	-0.18 – 0.06	0.356
Age (35-44)	-0.12	-2.12	-0.23 – -0.01	0.034
Age (45-54)	-0.18	-3.15	-0.29 – -0.07	0.002
Age (55-64)	-0.29	-5.18	-0.40 – -0.18	<0.001
Age (65+)	-0.32	-5.35	-0.44 – -0.20	<0.001
Education (technical or vocational degree)	-0.16	-3.36	-0.25 – -0.06	0.001
Education (university degree)	-0.10	-2.39	-0.19 – -0.02	0.017
Treatment (low amplification):CRT	0.06	2.64	0.01 – 0.10	0.008
Treatment (high amplification):CRT	0.00	-0.16	-0.05 – 0.04	0.875
Treatment (correction with link):CRT	0.02	0.89	-0.02 – 0.06	0.373
Random effects	*Var.*		mR^2	0.04

Participants (intercept)	0.34	0.58	cR^2	0.48
False News Posts (intercept)	0.02	0.14		
Residual	0.43	0.66		
**Liking**				
Fixed effects	*B*	*t*	*95% CI*	*p*
(Intercept)	2.89	33.61	2.72 – 3.06	<0.001
Treatment (low amplification)	-0.18	-4.10	-0.27 – -0.10	<0.001
Treatment (high amplification)	-0.13	-2.97	-0.22 – -0.04	0.003
Treatment (correction with link)	-0.22	-4.84	-0.30 – -0.13	<0.001
CRT	-0.22	-9.94	-0.27 – -0.18	<0.001
Gender (male)	0.22	5.07	0.13 – 0.30	<0.001
Age (25-34)	-0.07	-0.85	-0.24 – 0.10	0.396
Age (35-44)	-0.3	-3.73	-0.45 – -0.14	<0.001
Age (45-54)	-0.54	-6.77	-0.69 – -0.38	<0.001
Age (55-64)	-0.69	-8.77	-0.85 – -0.54	<0.001
Age (65+)	-0.64	-7.61	-0.81 – -0.48	<0.001
Education (technical or vocational degree)	-0.28	-4.29	-0.41 – -0.15	<0.001
Education (university degree)	-0.17	-2.85	-0.29 – -0.05	0.004
Treatment (low amplification):CRT	0.06	2.42	0.01 – 0.10	0.015
Treatment (high amplification):CRT	0.02	1.04	-0.02 – 0.07	0.296
Treatment (correction with link):CRT	0.05	2.35	0.01 – 0.10	0.019
Random effects	*Var.*		mR^2	0.10
Participants (intercept)	0.79	0.89	cR^2	0.67
False News Posts (intercept)	0.00	0.00		
Residual	0.46	0.68		
**Sharing**				
Fixed effects	*B*	*t*	*95% CI*	*p*
(Intercept)	2.96	33.95	2.79 – 3.13	<0.001
Treatment (low amplification)	-0.17	-4.14	-0.25 – -0.09	<0.001
Treatment (high amplification)	-0.11	-2.71	-0.19 – -0.03	0.007
Treatment (correction with link)	-0.19	-4.59	-0.27 – -0.11	<0.001
CRT	-0.23	-10.40	-0.28 – -0.19	<0.001
Gender (male)	0.26	5.83	0.17 – 0.35	<0.001
Age (25-34)	-0.13	-1.39	-0.30 – 0.05	0.163
Age (35-44)	-0.35	-4.26	-0.52 – -0.19	<0.001
Age (45-54)	-0.65	-7.89	-0.81 – -0.49	<0.001
Age (55-64)	-0.82	-10.03	-0.99 – -0.66	<0.001
Age (65+)	-0.81	-9.21	-0.98 – -0.64	<0.001
Education (technical or vocational degree)	-0.29	-4.29	-0.42 – -0.16	<0.001
Education (university degree)	-0.20	-3.09	-0.32 – -0.07	0.002
Treatment (low amplification):CRT	0.06	2.80	0.02 – 0.10	0.005
Treatment (high amplification):CRT	0.02	0.78	-0.03 – 0.06	0.436
Treatment (correction with link):CRT	0.05	2.19	0.00 – 0.09	0.029
Random effects	*Var.*		mR^2	0.12

Participants (intercept)	0.90	0.95	cR^2	0.74
False News Posts (intercept)	0.00	0.00		
Residual	0.38	0.62		

Supplementary Table 26. Results of linear mixed-effects models. The moderating role of anti-expert sentiments ($RQ_{\text{Anti-expert}}$) in the case of true news.

**Accuracy**				
Fixed effects	*B*	*t*	*95% CI*	*p*
(Intercept)	2.79	32.16	2.62 – 2.96	<0.001
Treatment (high amplification without cue)	-0.04	-0.67	-0.15 – 0.08	0.501
Treatment (high amplification with cue)	-0.18	-2.97	-0.29 – -0.06	0.003
Treatment (miscorrection with link)	-0.08	-1.41	-0.20 – 0.03	0.159
Anti-expert sentiments	-0.06	-3.52	-0.09 – -0.03	<0.001
Gender (male)	0.04	1.61	-0.01 – 0.08	0.107
Age (25-34)	0.04	0.81	-0.05 – 0.12	0.419
Age (35-44)	0.00	0.12	-0.08 – 0.09	0.904
Age (45-54)	0.00	0.03	-0.08 – 0.08	0.98
Age (55-64)	0.00	-0.12	-0.08 – 0.08	0.905
Age (65+)	-0.04	-0.85	-0.12 – 0.05	0.394
Education (technical or vocational degree)	0.11	3.31	0.04 – 0.17	0.001
Education (university degree)	0.07	2.34	0.01 – 0.13	0.019
Treatment (high amplification without cue):Anti-expert sentiments	-0.02	-0.99	-0.06 – 0.02	0.323
Treatment (high amplification with cue):Anti-expert sentiments	0.01	0.64	-0.03 – 0.05	0.521
Treatment (miscorrection with link):Anti-expert sentiments	-0.02	-0.92	-0.06 – 0.02	0.359
Random effects	*Var.*		mR^2	0.01
Participants (intercept)	0.17	0.41	cR^2	0.32
False News Posts (intercept)	0.05	0.22		
Residual	0.48	0.69		
**Liking**				
Fixed effects	*B*	*t*	*95% CI*	*p*
(Intercept)	2.1	19.07	1.89 – 2.32	<0.001
Treatment (high amplification without cue)	-0.06	-0.91	-0.19 – 0.07	0.362
Treatment (high amplification with cue)	-0.18	-2.65	-0.31 – -0.05	0.008
Treatment (miscorrection with link)	-0.09	-1.32	-0.22 – 0.04	0.187
Anti-expert sentiments	0.20	7.80	0.15 – 0.25	<0.001
Gender (male)	0.17	4.17	0.09 – 0.25	<0.001
Age (25-34)	-0.06	-0.73	-0.22 – 0.10	0.468
Age (35-44)	-0.26	-3.50	-0.41 – -0.12	<0.001
Age (45-54)	-0.48	-6.36	-0.62 – -0.33	<0.001
Age (55-64)	-0.49	-6.59	-0.64 – -0.35	<0.001
Age (65+)	-0.47	-5.90	-0.63 – -0.31	<0.001

Education (technical or vocational degree)	-0.28	-4.6	-0.40 – -0.16	<0.001
Education (university degree)	-0.19	-3.38	-0.31 – -0.08	0.001
Treatment (high amplification without cue):Anti-expert sentiments	0.00	0.22	-0.04 – 0.05	0.826
Treatment (high amplification with cue):Anti-expert sentiments	0.04	1.71	-0.01 – 0.08	0.088
Treatment (miscorrection with link):Anti-expert sentiments	0.01	0.34	-0.04 – 0.05	0.734
Random effects	*Var.*		*mR²*	0.06
Participants (intercept)	0.75	0.87	*cR²*	0.60
False News Posts (intercept)	0.02	0.14		
Residual	0.58	0.76		
**Sharing**				
Fixed effects	*B*	*t*	*95% CI*	*p*
(Intercept)	2.05	18.61	1.83 – 2.27	<0.001
Treatment (high amplification without cue)	-0.14	-2.32	-0.25 – -0.02	0.02
Treatment (high amplification with cue)	-0.21	-3.48	-0.33 – -0.09	<0.001
Treatment (miscorrection with link)	-0.16	-2.78	-0.28 – -0.05	0.005
Anti-expert sentiments	0.22	8.81	0.17 – 0.27	<0.001
Gender (male)	0.20	4.86	0.12 – 0.28	<0.001
Age (25-34)	-0.14	-1.61	-0.30 – 0.03	0.108
Age (35-44)	-0.35	-4.51	-0.50 – -0.20	<0.001
Age (45-54)	-0.61	-7.82	-0.76 – -0.45	<0.001
Age (55-64)	-0.66	-8.56	-0.81 – -0.51	<0.001
Age (65+)	-0.67	-8.1	-0.83 – -0.51	<0.001
Education (technical or vocational degree)	-0.27	-4.24	-0.39 – -0.14	<0.001
Education (university degree)	-0.20	-3.42	-0.32 – -0.09	0.001
Treatment (high amplification without cue):Anti-expert sentiments	0.02	1.17	-0.02 – 0.06	0.244
Treatment (high amplification with cue):Anti-expert sentiments	0.04	2.23	0.01 – 0.08	0.026
Treatment (miscorrection with link):Anti-expert sentiments	0.04	1.79	-0.00 – 0.07	0.073
Random effects	*Var.*		*mR²*	0.09
Participants (intercept)	0.83	0.91	*cR²*	0.68
False News Posts (intercept)	0.02	0.14		
Residual	0.47	0.69		

Supplementary Table 27. Results of linear mixed-effects models. The moderating role of susceptibility to social influence ($RQ_{Social\ influence}$) in the case of true news.

**Accuracy**				
Fixed effects	*B*	*t*	*95% CI*	*p*
(Intercept)	2.55	30.68	2.39 – 2.72	<0.001

Treatment (high amplification without cue)	-0.13	-3.07	-0.22 – -0.05	0.002
Treatment (high amplification with cue)	-0.21	-4.80	-0.29 – -0.12	<0.001
Treatment (amplification + link)	-0.23	-5.37	-0.31 – -0.15	<0.001
SSI	0.02	1.15	-0.01 – 0.05	0.251
Gender (male)	0.03	1.19	-0.02 – 0.07	0.235
Age (25-34)	0.03	0.78	-0.05 – 0.12	0.437
Age (35-44)	0.01	0.34	-0.07 – 0.10	0.736
Age (45-54)	0.02	0.60	-0.06 – 0.11	0.55
Age (55-64)	0.03	0.82	-0.05 – 0.12	0.412
Age (65+)	0.01	0.23	-0.08 – 0.10	0.821
Education (technical or vocational degree)	0.08	2.65	0.02 – 0.15	0.008
Education (university degree)	0.13	4.00	0.07 – 0.20	<0.001
Treatment (high amplification without cue):SSI	0.03	1.66	-0.01 – 0.06	0.097
Treatment (high amplification with cue):SSI	0.02	0.94	-0.02 – 0.05	0.349
Treatment (miscorrection with link):SSI	0.04	2.44	0.01 – 0.08	0.015
Random effects	*Var.*		*mR²*	0.01
Participants (intercept)	0.17	0.41	*cR²*	0.32
False News Posts (intercept)	0.05	0.22		
Residual	0.48	0.69		
**Liking**				
Fixed effects	*B*	*t*	*95% CI*	*p*
(Intercept)	1.33	13.51	1.13 – 1.52	<0.001
Treatment (high amplification without cue)	-0.08	-1.63	-0.17 – 0.02	0.103
Treatment (high amplification with cue)	-0.15	-3.23	-0.25 – -0.06	0.001
Treatment (miscorrection with link)	-0.11	-2.35	-0.20 – -0.02	0.019
SSI	0.44	20.47	0.40 – 0.48	<0.001
Gender (male)	0.16	4.46	0.09 – 0.23	<0.001
Age (25-34)	0.06	0.81	-0.08 – 0.20	0.416
Age (35-44)	-0.08	-1.22	-0.22 – 0.05	0.221
Age (45-54)	-0.16	-2.39	-0.30 – -0.03	0.017
Age (55-64)	-0.07	-1.07	-0.21 – 0.06	0.285
Age (65+)	0.00	0.04	-0.14 – 0.15	0.971
Education (technical or vocational degree)	-0.10	-1.99	-0.20 – -0.00	0.047
Education (university degree)	-0.16	-3.01	-0.27 – -0.06	0.003
Treatment (high amplification without cue):SSI	0.04	2.01	0.00 – 0.08	0.044
Treatment (high amplification with cue):SSI	0.01	0.75	-0.02 – 0.05	0.453
Treatment (miscorrection with link):SSI	0.02	1.07	-0.02 – 0.06	0.284
Random effects	*Var.*		*mR²*	0.18

Participants (intercept)	0.59	0.77	cR^2	0.60
False News Posts (intercept)	0.02	0.14		
Residual	0.58	0.76		
**Sharing**				
Fixed effects	*B*	*t*	*95% CI*	*p*
(Intercept)	1.19	12.32	1.00 – 1.38	<0.001
Treatment (high amplification without cue)	-0.07	-1.6	-0.15 – 0.02	0.111
Treatment (high amplification with cue)	-0.16	-3.62	-0.24 – -0.07	<0.001
Treatment (miscorrection with link)	-0.14	-3.39	-0.23 – -0.06	0.001
SSI	0.49	23.10	0.45 – 0.53	<0.001
Gender (male)	0.20	5.33	0.12 – 0.27	<0.001
Age (25-34)	0.00	-0.01	-0.15 – 0.15	0.992
Age (35-44)	-0.15	-2.17	-0.29 – -0.01	0.03
Age (45-54)	-0.26	-3.73	-0.40 – -0.12	<0.001
Age (55-64)	-0.20	-2.82	-0.34 – -0.06	0.005
Age (65+)	-0.15	-1.95	-0.30 – 0.00	0.052
Education (technical or vocational degree)	-0.10	-1.94	-0.21 – 0.00	0.052
Education (university degree)	-0.14	-2.55	-0.25 – -0.03	0.011
Treatment (low amplification):SSI	0.03	1.90	-0.00 – 0.07	0.057
Treatment (high amplification with cue):SSI	0.00	-0.07	-0.04 – 0.03	0.947
Treatment (miscorrection with link):SSI	0.04	2.10	0.00 – 0.07	0.036
Random effects	*Var.*		mR^2	0.23
Participants (intercept)	0.64	0.80	cR^2	0.68
False News Posts (intercept)	0.02	0.14		
Residual	0.47	0.69		

Supplementary Table 28. Results of linear mixed-effects models. The moderating role of cognitive reflection (RQ$_{\text{Cognitive reflection}}$) in the case of true news.

**Accuracy**				
Fixed effects	*B*	*t*	*95% CI*	*p*
(Intercept)	2.58	34.61	2.43 – 2.72	<0.001
Treatment (high amplification without cue)	0.04	2.82	0.01 – 0.06	0.005
Treatment (high amplification with cue)	-0.11	-3.65	-0.16 – -0.05	<0.001
Treatment (miscorrection with link)	-0.09	-3.29	-0.15 – -0.04	0.001
CRT	-0.12	-4.07	-0.17 – -0.06	<0.001
Gender (male)	0.02	0.83	-0.03 – 0.06	0.405
Age (25-34)	0.03	0.7	-0.06 – 0.12	0.484
Age (35-44)	0.01	0.21	-0.07 – 0.09	0.837
Age (45-54)	0.01	0.14	-0.07 – 0.09	0.892
Age (55-64)	0.01	0.18	-0.07 – 0.09	0.86
Age (65+)	-0.02	-0.48	-0.11 – 0.06	0.631
Education (technical or vocational degree)	0.06	2.03	0.00 – 0.13	0.043

Education (university degree)	0.10	3.03	0.04 – 0.17	0.002
Treatment (high amplification without cue):CRT	0.00	-0.04	-0.03 – 0.03	0.969
Treatment (high amplification with cue):CRT	-0.02	-1.58	-0.05 – 0.01	0.114
Treatment (miscorrection with link):CRT	-0.01	-0.76	-0.04 – 0.02	0.449
Random effects	*Var.*		*mR^2*	0.01
Participants (intercept)	0.18	0.42	*cR^2*	0.32
False News Posts (intercept)	0.05	0.22		
Residual	0.48	0.69		
**Liking**				
Fixed effects	*B*	*t*	*95% CI*	*p*
(Intercept)	2.83	32.61	2.66 – 3.00	<0.001
Treatment (high amplification without cue)	-0.05	-1.58	-0.11 – 0.01	0.113
Treatment (high amplification with cue)	-0.06	-1.88	-0.12 – 0.00	0.06
Treatment (amplification + link)	-0.05	-1.69	-0.12 – 0.01	0.091
CRT	-0.15	-7.67	-0.19 – -0.11	<0.001
Gender (male)	0.25	6.09	0.17 – 0.33	<0.001
Age (25-34)	-0.05	-0.59	-0.21 – 0.11	0.558
Age (35-44)	-0.29	-3.81	-0.44 – -0.14	<0.001
Age (45-54)	-0.50	-6.67	-0.65 – -0.35	<0.001
Age (55-64)	-0.55	-7.28	-0.70 – -0.40	<0.001
Age (65+)	-0.53	-6.59	-0.69 – -0.37	<0.001
Education (technical or vocational degree)	-0.14	-2.39	-0.25 – -0.03	0.017
Education (university degree)	-0.21	-3.48	-0.34 – -0.09	0.001
Treatment (high amplification without cue):CRT	0.01	0.65	-0.02 – 0.04	0.514
Treatment (high amplification with cue):CRT	-0.01	-0.61	-0.04 – 0.02	0.540
Treatment (miscorrection with link):CRT	-0.01	-0.36	-0.04 – 0.03	0.718
Random effects	*Var.*		*mR^2*	0.06
Participants (intercept)	0.76	0.87	*cR^2*	0.60
False News Posts (intercept)	0.02	0.14		
Residual	0.58	0.76		
**Sharing**				
Fixed effects	*B*	*t*	*95% CI*	*p*
(Intercept)	1.19	12.32	1.00 – 1.38	<0.001
Treatment (high amplification without cue)	-0.16	-3.62	-0.24 – -0.07	<0.001
Treatment (high amplification with cue)	-0.07	-1.60	-0.15 – 0.02	0.111
Treatment (miscorrection with link)	-0.14	-3.39	-0.23 – -0.06	0.001
CRT	0.49	23.10	0.45 – 0.53	<0.001
Gender (male)	0.20	5.33	0.12 – 0.27	<0.001
Age (25-34)	0.00	-0.01	-0.15 – 0.15	0.992

Age (35-44)	-0.15	-2.17	-0.29 – -0.01	0.030
Age (45-54)	-0.26	-3.73	-0.40 – -0.12	<0.001
Age (55-64)	-0.20	-2.82	-0.34 – -0.06	0.005
Age (65+)	-0.15	-1.95	-0.30 – 0.00	0.052
Education (technical or vocational degree)	-0.10	-1.94	-0.21 – 0.00	0.052
Education (university degree)	-0.14	-2.55	-0.25 – -0.03	0.011
Treatment (high amplification without cue):CRT	0.00	-0.07	-0.04 – 0.03	0.947
Treatment (high amplification with cue):CRT	0.03	1.90	-0.00 – 0.07	0.057
Treatment (miscorrection with link):CRT	0.04	2.10	0.00 – 0.07	0.036
Random effects	*Var.*		*mR*^{2}	0.23
Participants (intercept)	0.64	0.80	*cR*^{2}	0.68
False News Posts (intercept)	0.02	0.14		
Residual	0.47	0.69		

Note. The mixed-effects regressions for false news for $RQ_{Anti-expert}$ were run on 6,599 observations (perceived accuracy), 6,601 observations (like), and 6,601 observations (share) with 2,201 respondents and seven false news posts. The mixed-effects regressions for $RQ_{Social\ influence}$ were run on 6,602 observations (perceived accuracy), 6,604 observations (like), and 6,604 observations (share) with 2,202 respondents and seven false news posts. The mixed-effects regressions for $RQ_{Cognitive\ reflection}$ were run on 6,602 observations (perceived accuracy), 6,604 observations (like), and 6,604 observations (share) with 2,202 respondents and seven false news posts.
The mixed-effects regressions for true news for $RQ_{Anti-expert}$ were run on 13,201 observations (perceived accuracy), 13,202 observations (like), and 13,202 observations (share) with 2,202 respondents and 14 true news posts. The mixed-effects regressions for $RQ_{Social\ influence}$ were run on 13,207 observations (perceived accuracy), 13,208 observations (like), and 13,208 observations (share) with 2,203 respondents and 14 true news posts. The mixed-effects regressions for $RQ_{Cognitive\ reflection}$ were run on 13,207 observations (perceived accuracy), 13,208 observations (like), and 13,208 observations (share) with 2,203 respondents and 14 true news posts.

Supplementary Table 29. Results of linear mixed-effects models for $H_{Correct\ false}$ in Germany excluding respondents that failed attention checks.

**Perceived accuracy**				
Fixed effects	*B*	*t*	*95% CI*	*p*
(Intercept)	2.51	30.65	2.35 – 2.67	<0.001
Treatment (low amplification)	-0.11	4.11	-0.16 – -0.06	<0.001
Treatment (high amplification)	-0.14	5.31	-0.19 – -0.09	<0.001
Treatment (correction with link)	-0.15	5.83	-0.21 – -0.10	<0.001
Gender (male)	-0.01	0.44	-0.07 – 0.05	0.660
Age (25-34)	-0.08	-1.23	-0.21 – 0.05	0.219
Age (35-44)	-0.11	-1.79	-0.22 – 0.01	0.073
Age (45-54)	-0.16	-2.72	-0.27 – -0.04	0.007
Age (55-64)	-0.26	-4.45	-0.37 – -0.14	<0.001

Age (65+)	-0.28	-4.49	-0.40 – -0.16	<0.001
Education (technical or vocational degree)	-0.22	-4.67	-0.31 – -0.13	<0.001
Education (university degree)	-0.15	-3.26	-0.23 – -0.06	0.001
Random effects	*Variance*	*SD*	*mR*^{2}	0.02
Participants (intercept)	0.34	0.58	*cR*^{2}	0.47
False News Posts (intercept)	0.02	0.14		
Residual	0.43	0.66		
**Liking**				
Fixed effects	*B*	*t*	*95% CI*	*p*
(Intercept)	2.61	30.72	2.44 – 2.78	<0.001
Treatment (low amplification)	-0.10	-3.40	-0.15 – -0.04	0.001
Treatment (high amplification)	-0.10	-3.55	-0.15 – -0.04	<0.001
Treatment (correction with link)	-0.14	-4.83	-0.19 – -0.08	<0.001
Gender (male)	0.14	3.18	0.05 – 0.23	0.001
Age (25-34)	-0.10	-1.13	-0.28 – 0.08	0.257
Age (35-44)	-0.27	-3.18	-0.43 – -0.10	0.001
Age (45-54)	-0.51	-6.17	-0.67 – -0.35	<0.001
Age (55-64)	-0.63	-7.70	-0.80 – -0.47	<0.001
Age (65+)	-0.57	-6.49	-0.74 – -0.40	<0.001
Education (technical or vocational degree)	-0.40	-5.94	-0.53 – -0.27	<0.001
Education (university degree)	-0.24	-3.84	-0.37 – -0.12	<0.001
Random effects	*Variance*	*SD*	*mR*^{2}	0.05
Participants (intercept)	0.82	0.91	*cR*^{2}	0.66
False News Posts (intercept)	0.00	0.00		
Residual	0.45	0.67		
**Sharing**				
Fixed effects	*B*	*t*	*95% CI*	*p*
(Intercept)	2.68	30.83	2.51 – 2.85	<0.001
Treatment (low amplification)	-0.09	-3.43	-0.14 – -0.04	0.001
Treatment (high amplification)	-0.09	-3.44	-0.14 – -0.04	0.001
Treatment (correction with link)	-0.11	-4.45	-0.16 – -0.06	<0.001
Gender (male)	0.17	3.70	0.08 – 0.26	<0.001
Age (25-34)	-0.18	-1.92	-0.37 – 0.00	0.055
Age (35-44)	-0.34	-3.96	-0.51 – -0.17	<0.001
Age (45-54)	-0.63	-7.32	-0.79 – -0.46	<0.001
Age (55-64)	-0.76	-8.97	-0.93 – -0.60	<0.001
Age (65+)	-0.73	-8.09	-0.91 – -0.55	<0.001
Education (technical or vocational degree)	-0.41	-5.88	-0.54 – -0.27	<0.001
Education (university degree)	-0.28	-4.20	-0.40 – -0.15	<0.001
Random effects	*Variance*	*SD*	*mR*^{2}	0.07
Participants (intercept)	0.91	0.95	*cR*^{2}	0.73
False News Posts (intercept)	0.00	0.00		
Residual	0.37	0.61		

Note. The mixed-effects regressions were run on 6,345 observations (perceived accuracy), 6,347 observations (like), and 6,346 observations (share) with 2,116 respondents and 7 false news posts. *mR*^{2} = marginal R^2 (i.e., variance of the fixed effects); *cR*^{2} = conditional R^2 (i.e., variance of the fixed and random effects). Reference groups: Treatment = control condition; gender = female; age = 18-24; education = less than primary education.

Supplementary Table 30. Results of linear mixed-effects models for $H_{Correct\ false}$ in Germany controlling for congeniality.

Perceived accuracy				
Fixed effects	*B*	*t*	*95% CI*	*p*
(Intercept)	2.49	31.09	2.34 – 2.65	<0.001
Treatment (low amplification)	-0.10	-3.91	-0.15 – -0.05	<0.001
Treatment (high amplification)	-0.14	-5.52	-0.19 – -0.09	<0.001
Treatment (correction with link)	-0.16	-6.02	-0.21 – -0.11	<0.001
Gender (male)	-0.01	-0.38	-0.07 – 0.05	0.708
Age (25-34)	-0.04	-0.68	-0.17 – 0.08	0.497
Age (35-44)	-0.09	-1.57	-0.20 – 0.02	0.116
Age (45-54)	-0.15	-2.69	-0.27 – -0.04	0.007
Age (55-64)	-0.26	-4.48	-0.37 – -0.14	<0.001
Age (65+)	-0.28	-4.65	-0.40 – -0.16	<0.001
Education (technical or vocational degree)	-0.22	-4.73	-0.31 – -0.13	<0.001
Education (university degree)	-0.14	-3.26	-0.23 – -0.06	0.001
Congeniality (not congenial)	0.05	2.00	0.00 – 0.10	0.045
Random effects	*Variance*	*SD*	mR^2	0.03
Participants (intercept)	0.35	0.59	cR^2	0.48
False News Posts (intercept)	0.02	0.14		
Residual	0.43	0.66		
**Liking**				
Fixed effects	*B*	*t*	*95% CI*	*p*
(Intercept)	2.60	30.93	2.44 – 2.77	<0.001
Treatment (low amplification)	-0.10	-3.59	-0.16 – -0.05	<0.001
Treatment (high amplification)	-0.09	-3.36	-0.15 – -0.04	0.001
Treatment (correction with link)	-0.13	-4.80	-0.19 – -0.08	<0.001
Gender (male)	0.14	3.28	0.06 – 0.23	0.001
Age (25-34)	-0.05	-0.52	-0.22 – 0.13	0.600
Age (35-44)	-0.24	-2.98	-0.40 – -0.08	0.003
Age (45-54)	-0.49	-6.07	-0.65 – -0.33	<0.001
Age (55-64)	-0.63	-7.78	-0.79 – -0.47	<0.001
Age (65+)	-0.57	-6.62	-0.74 – -0.40	<0.001
Education (technical or vocational degree)	-0.40	-6.14	-0.53 – -0.27	<0.001
Education (university degree)	-0.25	-4.02	-0.37 – -0.13	<0.001
Congeniality (not congenial)	0.04	1.61	-0.01 – 0.10	0.107
Random effects	*Variance*	*SD*	mR^2	0.06
Participants (intercept)	0.84	0.92	cR^2	0.67
False News Posts (intercept)	0.00	0.00		
Residual	0.46	0.68		
**Sharing**				
Fixed effects	*B*	*t*	*95% CI*	*p*
(Intercept)	2.67	31.39	2.51 – 2.84	<0.001
Treatment (low amplification)	-0.08	-3.15	-0.13 – -0.03	0.002
Treatment (high amplification)	-0.08	-3.25	-0.13 – -0.03	0.001
Treatment (correction with link)	-0.12	-4.58	-0.17 – -0.07	<0.001
Gender (male)	0.18	4.00	0.09 – 0.27	<0.001
Age (25-34)	-0.10	-1.05	-0.28 – 0.08	0.294
Age (35-44)	-0.30	-3.48	-0.46 – -0.13	0.001

Age (45-54)	-0.60	-7.16	-0.77 – -0.44	<0.001
Age (55-64)	-0.76	-9.02	-0.93 – -0.59	<0.001
Age (65+)	-0.74	-8.20	-0.91 – -0.56	<0.001
Education (technical or vocational degree)	-0.42	-6.19	-0.56 – -0.29	<0.001
Education (university degree)	-0.28	-4.28	-0.40 – -0.15	<0.001
Congeniality (not congenial)	0.02	0.84	-0.03 – 0.07	0.399
Random effects	*Variance*	*SD*	*mR²*	0.08
Participants (intercept)	0.95	0.97	*cR²*	0.74
False News Posts (intercept)	0.00	0.00		
Residual	0.38	0.62		

Note. The mixed-effects regressions were run on 6,598 observations (perceived accuracy), 6,600 observations (like), and 6,600 observations (share) with 2,202 respondents and 7 false news posts. mR^2 = marginal R^2 (i.e., variance of the fixed effects); cR^2 = conditional R^2 (i.e., variance of the fixed and random effects). Reference groups: Treatment = control condition; gender = female; age = 18-24; education = less than primary education.

Supplementary Table 31. Results of linear mixed-effects models for $H_{\text{Miscorrect true}}$ in Germany excluding respondents that failed attention checks.

**Perceived accuracy**				
Fixed effects	*B*	*t*	*95% CI*	*p*
(Intercept)	2.64	35.47	2.50 – 2.79	<0.001
Treatment (high amplification without cue)	-0.10	-5.26	-0.13 – -0.06	<0.001
Treatment (high amplification with cue)	-0.14	-7.77	-0.18 – -0.11	<0.001
Treatment (miscorrection with link)	-0.14	-7.77	-0.18 – -0.11	<0.001
Gender (male)	0.04	1.75	-0.00 – 0.08	0.080
Age (25-34)	0.02	0.37	-0.07 – 0.11	0.711
Age (35-44)	-0.04	-0.92	-0.12 – 0.04	0.356
Age (45-54)	-0.03	-0.71	-0.11 – -0.05	0.477
Age (55-64)	-0.02	-0.58	-0.11 – 0.06	0.562
Age (65+)	-0.05	-1.21	-0.14 – -0.03	0.226
Education (technical or vocational degree)	0.12	3.60	-0.06 – -0.19	<0.001
Education (university degree)	0.08	2.42	0.01 – -0.14	0.016
Random effects	*Variance*	*SD*	*mR²*	0.01
Participants (intercept)	0.17	0.59	*cR²*	0.32
Real News Posts (intercept)	0.05	0.14		
Residual	0.48	0.66		
**Liking**				
Fixed effects	*B*	*t*	*95% CI*	*p*
(Intercept)	2.68	30.78	2.51 – 2.85	<0.001
Treatment (high amplification without cue)	-0.05	-2.48	-0.09 – -0.01	0.013
Treatment (high amplification with cue)	-0.07	-3.59	-0.11 – -0.03	<0.001
Treatment (miscorrection with link)	-0.07	-3.56	-0.11 – -0.03	<0.001
Gender (male)	0.19	4.56	0.11 – 0.27	<0.001
Age (25-34)	-0.09	-1.00	-0.26 – 0.08	0.315
Age (35-44)	-0.31	-3.90	-0.46 – -0.15	<0.001
Age (45-54)	-0.52	-6.65	-0.67 – -0.37	<0.001
Age (55-64)	-0.53	-6.82	-0.68 – -0.38	<0.001

Age (65+)	-0.49	-5.98	-0.65 – -0.33	<0.001
Education (technical or vocational degree)	-0.30	-4.77	-0.42 – -0.18	<0.001
Education (university degree)	-0.19	-3.12	-0.30 – -0.07	0.002
Random effects	*Variance*	*SD*	mR^2	0.04
Participants (intercept)	0.76	0.92	cR^2	0.59
Real News Posts (intercept)	0.03	0.00		
Residual	0.58	0.68		
**Sharing**				
Fixed effects	*B*	*t*	*95% CI*	*p*
(Intercept)	2.68	30.81	2.51 – 2.86	<0.001
Treatment (high amplification without cue)	-0.07	-3.96	-0.11 – -0.04	<0.001
Treatment (high amplification with cue)	-0.09	-4.77	-0.12 – -0.05	<0.001
Treatment (miscorrection with link)	-0.07	-3.81	-0.17 – -0.07	<0.001
Gender (male)	0.22	5.18	0.14 – 0.31	<0.001
Age (25-34)	-0.15	-1.69	-0.33 – 0.02	0.090
Age (35-44)	-0.38	-4.61	-0.54 – -0.22	<0.001
Age (45-54)	-0.63	-7.75	-0.78 – -0.47	<0.001
Age (55-64)	-0.67	-8.37	-0.83 – -0.52	<0.001
Age (65+)	-0.67	-7.81	-0.84 – -0.50	<0.001
Education (technical or vocational degree)	-0.30	-4.54	-0.42 – -0.17	<0.001
Education (university degree)	-0.21	-3.39	-0.33 – -0.09	0.001
Random effects	*Variance*	*SD*	mR^2	0.05
Participants (intercept)	0.85	0.97	cR^2	0.67
Real News Posts (intercept)	0.02	0.00		
Residual	0.46	0.62		

Note. The mixed-effects regressions were run on 6,602 observations (perceived accuracy), 6,604 observations (like), and 6,604 observations (share) with 2,202 respondents and 7 true news posts. mR^2 = marginal R^2 (i.e., variance of the fixed effects); cR^2 = conditional R^2 (i.e., variance of the fixed and random effects). Reference groups: Treatment = control condition; gender = female; age = 18-24; education = less than primary education.

Supplementary Table 32. Results of linear mixed-effects models for $H_{\text{Miscorrect true}}$ in Germany controlling for congeniality.

**Perceived accuracy**				
Fixed effects	*B*	*t*	*95% CI*	*p*
(Intercept)	2.59	34.65	2.45 – 2.74	<0.001
Treatment (high amplification without cue)	-0.10	-5.19	-0.13 – -0.06	<0.001
Treatment (high amplification with cue)	-0.14	-7.78	-0.18 – -0.11	<0.001
Treatment (miscorrection with link)	-0.14	-7.32	-0.17 – -0.10	<0.001
Gender (male)	0.03	1.42	-0.01 – 0.08	0.156
Age (25-34)	0.04	0.79	-0.05 – 0.12	0.429
Age (35-44)	0.01	0.19	-0.07 – 0.09	0.853
Age (45-54)	0.01	0.19	-0.07 – 0.09	0.849
Age (55-64)	0.00	0.11	-0.08 – 0.09	0.911
Age (65+)	-0.02	-0.55	-0.11 – -0.06	0.585
Education (technical or vocational degree)	0.11	3.37	0.05 – 0.18	0.001
Education (university degree)	0.07	2.14	0.03 – 0.13	0.033

Congeniality (not congenial)	0.04	1.85	-0.00 – 0.08	0.064
Random effects	*Variance*	*SD*	*mR^2*	0.01
Participants (intercept)	0.18	0.59	*cR^2*	0.32
Real News Posts (intercept)	0.05	0.14		
Residual	0.49	0.66		
**Liking**				
Fixed effects	*B*	*t*	*95% CI*	*p*
(Intercept)	2.63	30.42	2.46 – 2.80	<0.001
Treatment (high amplification without cue)	-0.05	-2.48	-0.09 – -0.01	0.013
Treatment (high amplification with cue)	-0.07	-3.49	-0.11 – -0.03	<0.001
Treatment (miscorrection with link)	-0.07	-3.54	-0.11 – -0.03	<0.001
Gender (male)	0.20	4.76	0.12 – 0.28	<0.001
Age (25-34)	-0.02	-0.30	-0.19 – 0.14	0.768
Age (35-44)	-0.24	-3.17	-0.40 – -0.09	0.002
Age (45-54)	-0.47	-6.09	-0.62 – -0.32	<0.001
Age (55-64)	-0.50	-6.48	-0.65 – -0.35	<0.001
Age (65+)	-0.47	-5.78	-0.63 – -0.31	<0.001
Education (technical or vocational degree)	-0.32	-5.20	-0.44 – -0.20	<0.001
Education (university degree)	-0.21	-3.52	-0.32 – -0.09	<0.001
Congeniality (not congenial)	0.06	2.30	0.01 – 0.10	0.021
Random effects	*Variance*	*SD*	*mR^2*	0.04
Participants (intercept)	0.79	0.89	*cR^2*	0.60
Real News Posts (intercept)	0.02	0.14		
Residual	0.58	0.76		
**Sharing**				
Fixed effects	*B*	*t*	*95% CI*	*p*
(Intercept)	2.64	30.59	2.47 – 2.81	<0.001
Treatment (high amplification without cue)	-0.07	-4.03	-0.11 – -0.04	<0.001
Treatment (high amplification with cue)	-0.09	-4.63	-0.12 – -0.05	<0.001
Treatment (miscorrection with link)	-0.07	-3.82	-0.11 – -0.03	<0.001
Gender (male)	0.23	5.40	0.15 – 0.32	<0.001
Age (25-34)	-0.09	-1.05	-0.26 – 0.08	0.296
Age (35-44)	-0.33	-4.04	-0.48 – -0.17	<0.001
Age (45-54)	-0.59	-7.44	-0.75 – -0.44	<0.001
Age (55-64)	-0.67	-8.33	-0.82 – -0.51	<0.001
Age (65+)	-0.67	-7.91	-0.84 – -0.51	<0.001
Education (technical or vocational degree)	-0.32	-4.95	-0.45 – -0.19	<0.001
Education (university degree)	-0.22	-3.59	-0.34 – -0.10	<0.001
Congeniality (not congenial)	0.07	3.21	0.03 – 0.11	0.001
Random effects	*Variance*	*SD*	*mR^2*	0.06
Participants (intercept)	0.89	0.94	*cR^2*	0.68
Real News Posts (intercept)	0.02	0.14		
Residual	0.47	0.69		

Note. The mixed-effects regressions were run on 12,851 observations (perceived accuracy), 12,851 observations (like), and 12,851 observations (share) with 2,203 respondents and 14 real news posts. mR^2 = marginal R^2 (i.e., variance of the fixed effects); cR^2 = conditional R^2 (i.e., variance of the fixed and random effects). Reference groups: Treatment = control condition; gender = female; age = 18-24; education = less than primary education.

Supplementary Table 33. Overview of demographics of the Germany Experiment.

**Demographic Variable**	**Frequency**	**Percentage (%)**
Gender		
Male	1091	49.4
Female	1112	50.3
Non-binary / third gender / other	7	0.3
Age		
18-24	242	11.0
25-34	283	12.8
35-44	431	19.5
45-54	454	20.5
55-64	452	20.5
65+	348	15.7
Education		
Low	351	15.9
Mid	1070	48.4
High	789	35.7
Party		
AfD	306	13.8
CDU/CSU	485	21.9
SPD	439	19.9
FDP	162	7.3
Greens	310	14.0
Left	139	6.3
No PID	303	13.7
Other party	66	3.0

Note. Education: low = incomplete Secondary Education, Secondary Education Completed, or Secondary Education Completed; mid = some Vocational or Technical Qualifications or Vocational or Technical Qualifications Completed; high = University Education Completed (First Degree e.g., BA, BSc), Postgraduate Education Completed (e.g., Masters), Doctorate, or Post-doctorate or equivalent. Party: No PID = *no party* and *don't know* option. Note that "Other party" includes one NA.

Supplementary Table 34. Test of differences between conditions (UK, Italy, and Germany)

**False news**						
**UK**	***B***	***SE***	***t***	***df***	***p***	***95% CI***
Low amplification vs. high amplification	-0.03	0.02	-1.55	9833	0.121	-0.07 – 0.01
Low amplification vs. correction with link	-0.02	0.02	-0.92	9833	0.358	-0.05 – 0.02
High amplification vs. correction with link	0.01	0.02	0.62	9833	0.532	-0.03 – 0.05
**Italy**						
Low amplification vs. high amplification	-0.08	0.02	-4.49	10186	<0.001	-0.12 – -0.05
Low amplification vs. correction with link	-0.08	0.02	-4.35	10186	<0.001	-0.12 – -0.04
High amplification vs. correction with link	0.00	0.02	0.16	10186	0.870	-0.03 – 0.04
**Germany**						
Low amplification vs. high amplification	-0.04	0.03	-1.53	6587	0.127	-0.09 – 0.01
Low amplification vs. correction with link	-0.05	0.03	-2.09	6587	0.037	-0.11 – 0.00
High amplification vs. correction with link	0.04	0.03	1.53	6587	0.127	-0.01 – 0.09
**True news**						
**UK**						
Low amplification vs. high amplification	-0.12	0.03	-4.02	5794	<0.001	-0.18 – -0.06
Low amplification vs. correction with link	-0.04	0.03	-1.47	5794	0.141	-0.10 – 0.01
High amplification vs. correction with link	0.08	0.03	2.52	5794	0.012	-0.02 – 0.13
**Italy**						
Low amplification vs. high amplification	-0.04	0.02	-1.96	9761	0.050	-0.09 – 0.00
Low amplification vs. correction with link	0.01	0.02	0.37	9761	0.715	-0.04 – 0.05
High amplification vs. correction with link	0.05	0.02	2.33	9761	0.020	0.01 – 0.10
**Germany**						
High amplification (no cue) vs. high amplification (cue)	-0.05	0.02	-2.49	13192	0.013	-0.08 – 0.00
High amplification (no cue) vs. correction with link	0.01	0.02	0.37	13192	0.710	-0.03 – 0.04
High amplification (cue) vs. correction with link	-0.04	0.02	-2.13	13192	0.033	-0.07 – 0.00

Supplementary Table 35. Analysis with false and true news data pooled (accuracy perceptions) for UK, Italy, and Germany

UK				
Fixed effects	*B*	*t*	*95% CI*	*p*
(Intercept)	2.32	30.99	2.18 – 2.47	<0.001
News type (true)	0.43	3.38	0.18 – 0.67	0.001
Treatment (low amplification)	-0.10	-4.71	-0.14 – -0.06	<0.001
Treatment (high amplification)	-0.12	-6.13	-0.16 – -0.08	<0.001
Treatment (correction with link)	-0.11	-5.60	-0.15 – -0.07	<0.001
Gender (male)	0.10	3.55	0.04 – 0.15	<0.001
Age (25-34)	0.06	1.08	-0.05 – 0.16	0.281
Age (35-44)	-0.01	-0.23	-0.11 – 0.09	0.814
Age (45-54)	-0.33	-6.39	-0.43 – -0.23	<0.001
Age (55-64)	-0.45	-8.44	-0.55 – -0.34	<0.001
Age (65+)	-0.48	-9.55	-0.57 – -0.38	<0.001
Education (technical or vocational degree)	0.00	0.12	-0.06 – 0.07	0.907
Education (university degree)	0.05	1.64	-0.01 – 0.12	0.101
News type (true):Treatment (low amplification)	0.04	1.30	-0.02 – 0.11	0.193
News type (true):Treatment (high amplification)	-0.02	-0.55	-0.08 – 0.05	0.579
News type (true):Treatment (correction with link)	0.03	0.81	-0.04 – 0.09	0.419
Random effects	*Variance*	*SD*	mR^2	0.11
Participants (intercept)	0.31	0.56	cR^2	0.49
News Posts (intercept)	0.04	0.20		
Residual	0.46	0.68		

Italy				
Fixed effects	*B*	*t*	*95% CI*	*p*
(Intercept)	2.06	30.19	1.93 – 2.20	<0.001
News type (true)	0.52	5.34	0.33 – 0.71	<0.001
Treatment (low amplification)	-0.04	-1.97	-0.08 – -0.00	0.048
Treatment (high amplification)	-0.11	-5.40	-0.15 – -0.07	<0.001
Treatment (correction with link)	-0.12	-5.56	-0.16 – -0.07	<0.001
Gender (male)	-0.03	-1.17	-0.07 – 0.02	0.240
Age (25-34)	-0.08	-2.00	-0.17 – -0.00	0.046
Age (35-44)	-0.18	-4.67	-0.26 – -0.11	<0.001
Age (45-54)	-0.16	-4.07	-0.24 – -0.08	<0.001
Age (55-64)	-0.20	-4.89	-0.29 – -0.12	<0.001
Age (65+)	-0.20	-1.19	-0.53 – 0.13	0.234
Education (technical or vocational degree)	0.02	0.87	-0.03 – 0.08	0.385
Education (university degree)	0.04	1.08	-0.03 – 0.11	0.278
News type (true):Treatment (low amplification)	-0.03	-0.97	-0.09 – 0.03	0.334
News type (true):Treatment (high amplification)	0.01	0.18	-0.05 – 0.06	0.860
News type (true):Treatment (correction with link)	0.06	2.12	0.00 – 0.12	0.034

Random effects	*Variance*			*mR²*	0.09
Participants (intercept)	0.24	0.49		*cR²*	0.41
News Posts (intercept)	0.02	0.14			
Residual	0.49	0.7			
Germany					
Fixed effects	*B*	*t*	*95% CI*		*p*
(Intercept)	2.25	26.07	26.07		<0.001
News type (true)	0.51	5.24	5.24		<0.001
Treatment (low amplification)	-0.10	-3.60	-3.60		<0.001
Treatment (high amplification)	-0.14	-4.96	-4.96		<0.001
Treatment (correction with link)	-0.17	-6.09	-6.09		<0.001
Gender (male)	0.02	0.92	0.92		0.355
Age (25-34)	0.00	0.01	0.01		0.990
Age (35-44)	-0.03	-0.88	-0.88		0.379
Age (45-54)	-0.06	-1.62	-1.62		0.106
Age (55-64)	-0.10	-2.70	-2.70		0.007
Age (65+)	-0.12	-3.24	-3.24		0.001
Education (technical or vocational degree)	0.00	0.12	0.12		0.906
Education (university degree)	0.01	0.25	0.25		0.804
News type (true):Treatment (low amplification)	-0.05	-1.52	-1.52		0.129
News type (true):Treatment (high amplification)	0.04	1.06	1.06		0.287
News type (true):Treatment (correction with link)	0.03	0.82	0.82		0.410
Random effects	*Variance*	*SD*		*mR²*	0.08
Participants (intercept)	0.13	0.36		*cR²*	0.29
News Posts (intercept)	0.04	0.20			
Residual	0.58	0.76			

Supplementary Table 36: Overview of false news headlines shown on social media posts and links to debunking information

Country	Headline<br>*Translated to English for Italian and German headlines*	Debunk link
UK	There are only TWO projects in history that have cost more than the Track & Trace app: …	https://fullfact.org/online/track-and-trace-project-cost/
	So we're just not going to talk about the Delta pilot who died mid-flight…	https://fullfact.org/online/delta-airlines-pilot-died-mid-flight-false/
	Deaths among male Children [sic.] are 83% higher than the 5-year-average since they were given the Covid-19 Vaccine	https://www.reuters.com/article/factcheck-yeadon-children50times/fact-check-no-evidence-to-support-claim-by-ex-pfizer-scientist-on-covid-19-vaccine-safety-in-children-idUSL1N2S72HQ
	Did you know, another interesting point. Fishing boats run on EU subsidised fuel…	https://fullfact.org/online/Brexit-EU-fishing-fuel-subsidies/
	REPORT: A Group Of Diesel Gas Generators Are Hidden From Public View Behind Screens At The #COP26 Climate Conference. …	https://www.reuters.com/article/idUSL1N2S21ZB/<br><br>https://apnews.com/article/fact-checking-994848902455
	Dr. Michael Yeadon, former Pfizer president, warns children are 50 times more likely to be killed by the covid vaccines than the virus itself	https://www.reuters.com/article/factcheck-yeadon-children50times/fact-check-no-evidence-to-support-claim-by-ex-pfizer-scientist-on-covid-19-vaccine-safety-in-children-idUSL1N2S72HQ
	Myriam Bourla - the wife of Pfizer CEO Albert Bourla - has died from complications from the COVID-19 vaccine…	https://www.reuters.com/article/factcheck-pfizer-wife/fact-check-false-claim-about-pfizer-ceos-wife-dying-after-complications-from-covid-19-vaccine-idUSL1N2S72TC
	There are 400 private jets ferrying delegations to the COP26 conference. …	https://fullfact.org/environment/cop26-private-jets-scotland-carbon-emissions-year/
	US Biolabs in Ukraine; Exclusive US biolabs in Ukraine, and they are financed at the expense of the US Department of Defense	https://eu.usatoday.com/story/news/factcheck/2022/02/25/fact-check-claim-us-biolabs-ukraine-disinformation/6937923001/<br><br>https://www.bbc.com/news/60711705
	Classified documents (order by the Commander National Guard of Ukraine) acquired by @mod_russia confirm…	https://www.politifact.com/factchecks/2022/mar/14/tweets/no-document-doesnt-show-secret-ukrainian-military-/

Italy	In Italy, all bank accounts of Russian citizens are being blocked.	https://www.bufale.net/no-in-italia-non-stanno-bloccando-i-conti-correnti-ai-cittadini-russi-tra-bufala-e-confusione/
	Italy is already at war, the Army heads towards Russia's new campaign	https://www.bufale.net/no-litalia-non-e-gia-in-guerra-con-la-campagna-di-russia-fermiamo-i-titoloni/
	All the doctors who discovered cancer enzymes in vaccines are dead	https://www.bufale.net/i-medici-che-hanno-scoperto-gli-enzimi-del-cancro-nei-vaccini-trovati-tutti-assassinati/
	No-vaaax maaales; You are riich<br><br>"The price of sperm from unvaccinated people increases by 400%."	https://www.bufale.net/il-ritorno-della-bufala-dello-sperma-novax/
	News from Siena: Italians sleep in stations, while refugees in luxury housing	https://www.bufale.net/bufala-immigrati-in-hotel-lusso-italiani-homeless-siena/
	Artemisia: This herb kills 98% of a tumor in just 16 hours!	https://www.bufale.net/bufala-artemisia-questa-erba-uccide-tumore-in-sole-16-ore-bufale-net/
	KIDS ARE NOT ALLOWED TO DRAW THEIR MOM AND DAD. To avoid offending the children of homosexuals: ...	https://www.bufale.net/bufala-divieto-bambini-disegnare-mamma-papa-bufale-net/
	Angela Merkel: "Italy had a great opportunity to join the Euro, but being a country of clowns, they weren't able to take advantage of it."	https://www.bufale.net/bufala-merkel-gli-italiani-pagliacci/
	In Peru, they are dismantling 5G antennas	https://www.bufale.net/hanno-smantellato-unantenna-5g-in-peru-no-il-video-e-fuori-contesto/
Germany	Ginger kills 10'000-times more cancer cells than chemotherapy	https://leadstories.com/hoax-alert/2019/09/fake-news-study-does-not-find-ginger-10,000x-more-effective-at-killing-cancer-than-chemo.html<br><br>https://healthfeedback.org/claimreview/claim-that-ginger-is-more-effective-than-chemotherapy-for-cancer-treatment-is-unsupported/<br><br>https://www.medizin-transparent.at/brustkrebs-ingwer/

	Could there also be other political interests behind Covid-19 after all?	https://correctiv.org/faktencheck/2020/12/04/diese-grafik-zu-influenza-und-coronavirus-sterbefaellen-stellt-die-daten-selektiv-und-irrefuehrend-dar/
	Information about the prescribed "obligation to wear a mask in shops"	https://correctiv.org/faktencheck/2020/05/25/macht-maske-tragen-krank-irrefuehrende-behauptungen-im-umlauf/
	Real pandemic - staged pandemic; Everyone knows very ill people…	https://correctiv.org/faktencheck/2021/09/20/das-video-die-pandemie-in-rohdaten-laesst-kontext-aus-und-fuehrt-so-in-die-irre/<br><br>https://correctiv.org/faktencheck/hintergrund/2022/06/17/coronavirus-faktenchecks-diese-behauptungen-hat-correctiv-geprueft/
	Booster-warning! The more "vaccines" a person has injected against the Covid-19 virus, …	https://faktencheck.afp.com/doc.afp.com.32782HM
	5G and unpredictable consequences	https://correctiv.org/faktencheck/2020/08/28/keine-belege-dafuer-dass-5g-krank-macht/
	"Genetic information is destabilised"	https://correctiv.org/faktencheck/2020/09/11/nein-studie-belegt-nicht-dass-elektromagnetische-strahlung-wie-5g-krebs-erzeugt/
